# Radiotherapy induces YTHDF2 in dendritic cells impairing cross-presentation and T cell function

**DOI:** 10.1084/jem.20250641

**Published:** 2025-11-06

**Authors:** Dapeng Chen, Liangliang Wang, Chuangyu Wen, Andras Piffko, Jason Bugno, Xianbin Yu, Pingluan Wang, Fei Ji, Emile Z. Naccasha, Jiaai Wang, Xiaona Huang, Steven J. Chmura, Sean P. Pitroda, Chuan He, Hua Laura Liang, Ralph R. Weichselbaum

**Affiliations:** 1 Department of Radiation and Cellular Oncology, University of Chicago, Chicago, IL 60637, USA.; 2 Ludwig Center for Metastasis Research, University of Chicago, Chicago, IL 60637, USA.; 3 Department of Chemistry, Department of Biochemistry and Molecular Biology, and Institute for Biophysical Dynamics, University of Chicago, Chicago, IL 60637, USA; 4 Howard Hughes Medical Institute, University of Chicago, Chicago, IL 60637, USA; 5 The Laboratory of Microbiome and Microecological Technology, Institute of Microbiology, Chinese Academy of Sciences, Beijing, China.; 6 Department of Neurosurgery, University Medical Center Hamburg-Eppendorf, Hamburg, Germany.

## Abstract

Metastatic progression is a major cause of radiotherapy (RT) failure, yet the mechanisms linking RT to immune suppression and metastasis remain unclear. Here, we identify YTHDF2 as a radiation-induced immune checkpoint in dendritic cells (DCs). By analyzing patient biopsies from a clinical trial (NCT03223155), we discover that increased YTHDF2 expression in DCs post RT correlates with treatment failure after RT. Mechanistically, ionizing radiation (IR) induces SPI1, which drives transcription of *Ythdf2* in DCs. Upregulated YTHDF2 promotes m^6^A-mediated degradation of Notch pathway regulators (*Mfng*, *Aph1b*, *Aph1c*), impairing MHC-I cross-presentation and CD8^+^ T cell activation, thereby facilitating tumor immune evasion and metastatic spread. Crucially, targeting YTHDF2 restores DC immunogenicity, enhances RT-induced tumor control, and improves DC-based cancer vaccines when combined with radiotherapy, providing a clinically actionable strategy to overcome RT resistance and metastasis.

## Introduction

Radiotherapy (RT) is a widely used cancer treatment, but its effectiveness is often compromised by immune suppression and distant metastasis.(Wang et al., 2024a; Weichselbaum et al., 2017) While RT can enhance CD8^+^ T cell activation by promoting dendritic cell (DC)-mediated antigen presentation, it also triggers immunosuppressive pathways that weaken systemic anti-tumor immunity.(Demaria et al., 2004; Lee et al., 2009) RT has been shown to suppress DC type I interferon signaling,(Hou et al., 2018) induces DC production of immunoregulatory cytokine CCL22,(Bugno et al., 2024) and upregulate inhibitory molecules such as PD-L1 in DCs,(Hou et al., 2024) contributing to tumor progression and metastatic spread. Despite efforts to combine RT with immune checkpoint blockade (ICB), clinical trials have yielded limited success,(Bestvina et al., 2022; Spaas et al., 2023) highlighting the need for identifying new therapeutic targets in DCs to enhance RT efficacy and prevent metastatic escape.

RNA N^6^-methyladenosine (m^6^A) modifications are key regulators of immune function,(Chen et al., 2024; Dong et al., 2021; Ma et al., 2023; Su et al., 2020; Wang et al., 2023; Wang et al., 2024b; Xiao et al.) yet their roles in DC-driven anti-tumor immunity remains to be fully elucidated. Among m^6^A reader proteins, YTHDF1 enhances mRNA translation,(Wang et al., 2015) while YTHDF2 primarily promotes mRNA decay,(Wang et al., 2014) both playing distinct roles in immune regulation. YTHDF1 is a key regulator of antigen processing, as our previous work showed that YTHDF1-m^6^A machinery facilitates the translation of lysosomal cathepsins in DCs, leading to excessive degradation of ingested neoantigens and impaired anti-tumor immunity.(Han et al., 2019; Wen et al., 2024) In contrast, YTHDF2 plays a broader role in mRNA stability and has been implicated in critical biological processes such as cell cycle regulation,(Fei et al., 2020) stress response,(Yu et al., 2019) hematopoietic stem cell expansion,(Li et al., 2018b; Paris et al., 2019) and immune cell differentiation.(Zhang et al., 2024) However, its role in DC immunogenicity, RT response, and metastatic progression remains unknown.

Here, we identify YTHDF2 as a radiation-induced immune checkpoint in DCs that drives tumor immune evasion and metastatic progression. Analysis of patient biopsies from a clinical trial (NCT03223155) reveals that RT-induced YTHDF2 expression in DCs correlates with disease progression. We further identify the transcription factor regulating YTHDF2 expression after RT, define the role of YTHDF2 in DC-mediated anti-tumor immunity, and identify the target genes that control DC MHC-I antigen presentation. Notably, we demonstrate that pharmacologically blocking of YTHDF2 restores DC immunogenicity and enhances the efficacy of DC-based vaccines, offering a promising strategy to improve RT outcomes and prevent metastasis.

## Results

### Radiation induces YTHDF2 expression in DCs of patients with metastasis progression

**Figure 1.**
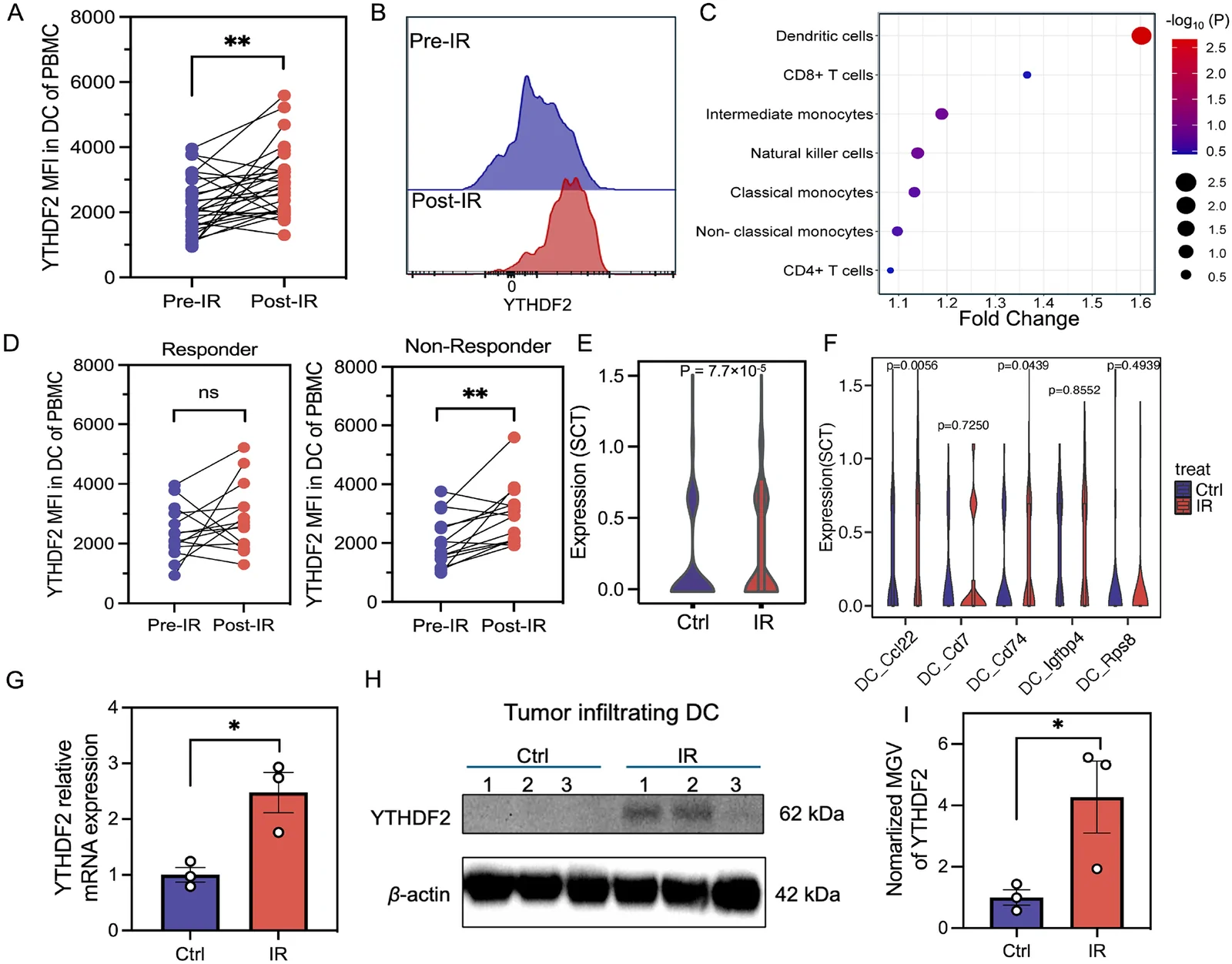
IR induces YTHDF2 expression in dendritic cells. (**A**) Mean fluorescent intensity (MFI) of YTHDF2 in DCs of PBMCs collected from lung cancer patients pre- and post-RT. The post-RT blood samples were collected approximately 1–3 weeks (median 2 weeks) after the collection of pre-RT samples, n = 28. (**B**) Representative histogram of YTHDF2 expression in DCs of patients presented in (A) pre-RT and post-RT. (**C**) Dot bubble plot of YTHDF2 level changes in DCs induced by IR across different types of immune cells in PBMCs of patients. (**D**) YTHDF2 level in DCs of paired PBMCs from responders (n = 14) and non-responder patients (n = 14). (**E**) Violin plot of YTHDF2 expression in MC38 tumor-infiltrating DCs of tumors treated by control or IR. (**F**) Violin plot of YTHDF2 expression across different clusters of tumor-infiltrating DCs of tumors treated by control or IR. (**G**) YTHDF2 mRNA expression by qPCR in DCs isolated from tumor received IR or control treatment, n = 3. (**H**) Western blotting of YTHDF2 protein in DCs isolated from MC38 tumor treated by control or IR. 1–3, number of repeats (n = 3). (**I**) Normalized mean grey value (MGV) of western blotting results in Figure 1H, n = 3. Statistical analysis was performed using two-sided paired Student’s t test (A, D) and unpaired Student’s t test (E-G, I); ns = not significant, P>0.05; *P<0.05; **P<0.01. Data are represented as mean ± standard error of mean (SEM), and n = number of samples. Source data are available for this figure: SourceData Fig1.

To explore the relationship between YTHDF2 expression and radiation, we quantified YTHDF2 expression in patients and murine models following irradiation. We first collected the peripheral blood mononuclear cells (PBMCs) prior to and following RT from patients treated on a phase I clinical trial (COSINR, NCT03223155) at our institution (Fig. S1A, Table S1).(Bestvina et al., 2022; Spurr et al., 2022) Patients received stereotactic body radiotherapy (SBRT) and either concurrent or sequential immunotherapy (ipilimumab and nivolumab) as a first-line treatment for metastatic non-small cell lung cancer (NSCLC). We performed spectral flow cytometry to examine YTHDF2 protein level in various immune cell populations from PBMCs of paired pre- and post-RT samples (Fig. S1A for gating strategy). We observed that the YTHDF2 expression level was significantly increased in DCs following IR (Fig. 1A and 1B) to a higher degree than other immune cells such as CD4^+^ T cells, CD8^+^ T cells and monocytes (Fig. 1C). To evaluate the correlation between induction of YTHDF2 expression by RT and tumor progression, we separated patients into responders who did not progress at distant sites after treatment and non-responders whose lesions progressed at distant sites.(Bestvina et al., 2022; Spurr et al., 2022) There was no significant changes of DC frequency between responders and non-responders pre- and post-IR (Fig. S1B and S1D). However, YTHDF2 protein level significantly increased following IR in non-responders but not in responders (Fig. 1D), indicating that YTHDF2 in DCs is associated with metastatic disease progression. We further analyzed YTHDF2 expression levels in patients prior to IR treatment, and we found no significant differences between responders and non-responders (Fig. S1F). This suggests that elevated YTHDF2 expression is not generally associated with disease progression, but more specifically linked to the radiotherapy-treated subgroup.

To explore the YTHDF2 of DCs in the tumor immune microenvironment (TIME), we characterized murine CD45^+^ immune cells from irradiated (4 days post-IR) and non-irradiated MC38 tumors *via* single-cell RNA sequencing (scRNA-seq). The average mRNA level of YTHDF2 significantly increased in tumor-infiltrating DCs after IR (Fig. 1E and S1G). We further identified five major subsets of DCs with markers of *Ccl22*, *Cd74*, *Cd7*, *Igfbp4*, and *Rps8* (Fig. S1C, S1E and S1H) and then characterized the changes within DC subtypes in irradiated tumors compared to non-irradiated tumors. We observed that YTHDF2 mRNA levels were significantly elevated in *Ccl22* DCs (mregDCs) and *Cd74* dendritic cells in tumors following IR (Fig. 1F and S1I). Next, we collected tumor-infiltrating DCs by gating live CD45+CD11c+MHC-II+Ly6C−F4/80− cells at day 5 post IR and confirmed that the YTHDF2 mRNA level in DCs increased after IR (Fig. 1G). The protein level of YTHDF2 was also up-regulated in tumor-infiltrating DCs treated with IR (Fig. 1H and 1I). When co-cultured with irradiated MC38-OT1-zsgreen (MC38-OZ) tumor cells, Flt3l-induced bone marrow DC (BMDCs) exhibited elevated YTHDF2 at both mRNA and protein levels compared to that of DCs cocultured with un-irradiated MC38-OZ cells (Fig. S1J–S1M). Direct irradiation (5 Gy) of BMDCs did not contribute to the induction of YTHDF2 (Fig. S1N). To investigate the tumor-derived signals that regulate *Ythdf2* in DCs, we compared co-cultures using: irradiated tumor cell-conditioned supernatant, and irradiated tumor cells with the medium removed. We found that both direct cell-cell contact and soluble factors contribute to *Ythdf2* induction (Fig. S1O), but the effect was over 3-fold greater in conditions with direct cell contact. Moreover, YTHDF2 expression is specifically increased in both cDC1 and cDC2 populations upon IR rather than in pDCs (Fig. S1P), supports that the effect observed in Fig. 1G is primarily driven by conventional DC subsets. Taken together, these results demonstrate that IR induces YTHDF2 expression in DCs in both clinical and pre-clinical settings and that YTHDF2 induction in DCs could play a role in metastatic cancer progression.

### SPI1 promotes transcription of *Ythdf2* in the context of IR

**Figure 2.**
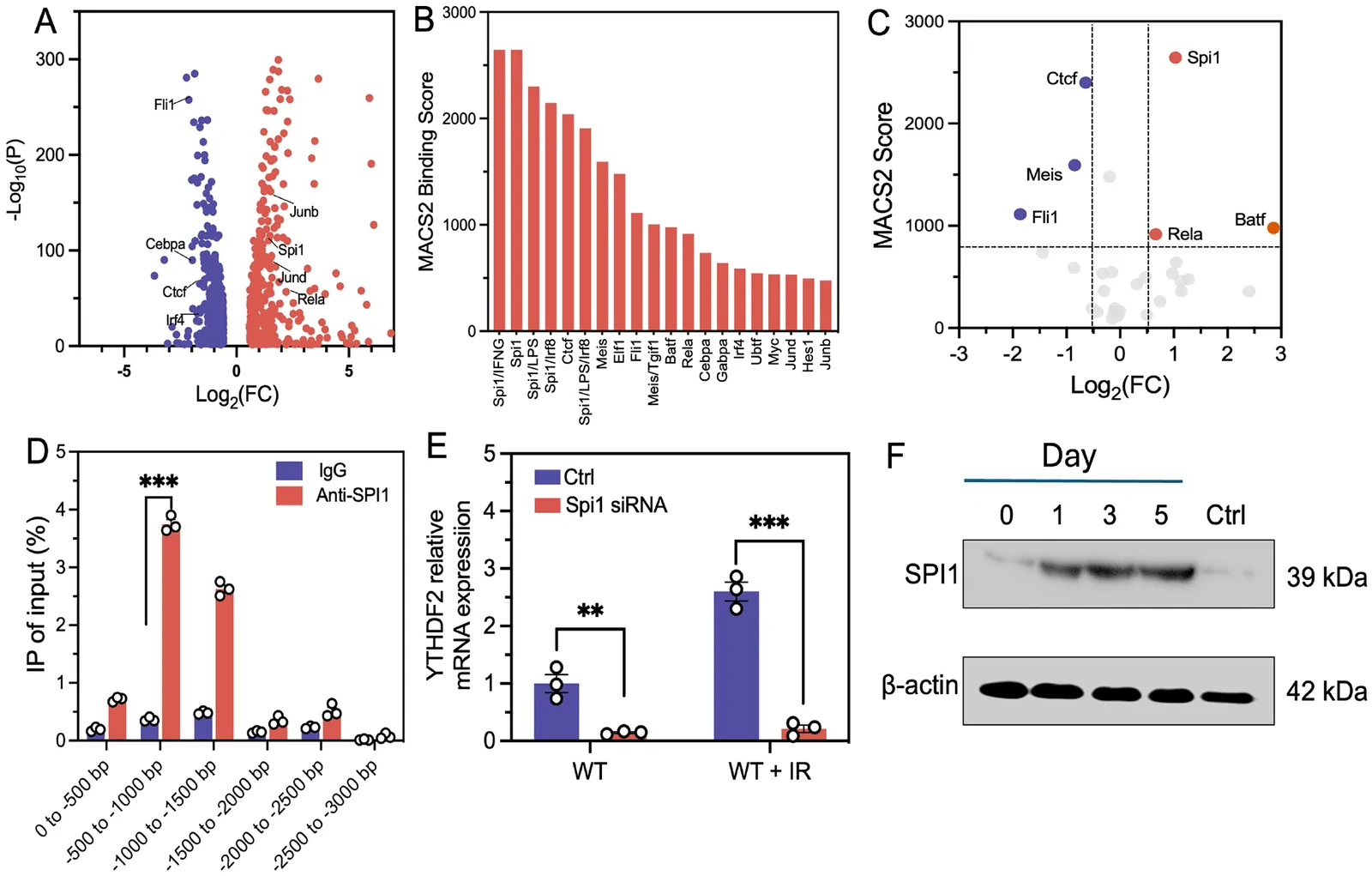
SPI1 promotes transcription of *Ythdf2* in the context of IR. (**A**) Volcano plot of differential expressed genes from upregulated (red) and downregulated (blue) transcription pathways of tumor-infiltrating DCs (WT+IR versus WT+control). (**B**) Bar chart showing the MACS2 score of top-20 ranked transcription factors binding to *Ythdf2*. A higher MACS2 score indicating the higher capability of TF binding to *Ythdf2*. (**C**) Integrated volcano plot of MACS2 score and differential expressed TF genes, upregulated (red) and downregulated (blue), for *Ythdf2* transcription in DCs. (WT+IR versus WT+control). (**D**) ChIP-qPCR results showing SPI1 binding to the promoter region of *Ythdf2* (n = 3). (**E**) *Ythdf2* mRNA expression in *Spi1* KD DCs co-cultured with control or irradiated tumor cells (n = 3). (**F**) SPI1 protein level in tumor-infiltrating DCs at different days post IR (20 Gy). Statistical analysis was performed using two-sided unpaired Student’s t test (D-E); **P <0.01; ***P<0.001. Data are represented as mean ± SEM. Source data are available for this figure: SourceData Fig2.

To investigate the potential mechanisms of YTHDF2 induction by IR, we analyzed tumor-infiltrating DCs collected 5 days post-IR using RNA-seq. Gene Ontology (GO) enrichment analysis indicated that IR impacted multiple biological processes (BP) of DCs, including upregulation (Fig. S2A) and downregulation of pathways (Fig. S2B) involved in transcriptional activities such as those regulated by various transcription factors (e.g. *Ctcf*, *Irf4*, *Spi1*, *Jund*, *Rela*) (Fig. 2A). In parallel, we used the cell signaling pathway project database(Ochsner et al., 2019) to evaluate protein binding to the *Ythdf2* promoter by assessing model-based analysis of ChIP-seq2 (MACS2) score. Several transcription factors (TFs), including SPI1, CTCF, MEIS, EIF and RELA, showed a top MACS2 score for *Ythdf2*, indicating potential binding TFs (Fig. 2B) Integrating the MACS2 score and RNA-seq output, we identified three TFs (SPI1, RELA, and BATF) as top candidates which could affect the transcription of *Ythdf2* (Fig. 2C).

To further examine the binding of these transcription factors to the YTHDF2 promoter in DCs following IR, we performed chromatin immunoprecipitation (ChIP) quantitative PCR using BMDCs. The results showed that SPI1 directly binds to the promoter region of *Ythdf2* (0.5–1.0 kb proximal to the transcription start site, Fig. 2D), whereas RELA and BATF also showed binding to *Ythdf2* region at 0–0.5 kb (Fig. S2C) and 0.5–1.0 kb (Fig. S2D), respectively. To study the time course of binding to the YTHDF2 promoter, we performed ChIP-qPCR for SPI1, RELA, and BATF binding at the *Ythdf2* promoter at multiple time points (0 h, 0.5 h, 1 h, 2 h, and 4 h) after IR. SPI1 binding peaked at 1–2 hours post-irradiation (Fig. S2E); BATF and RELA showed transient binding, peaking at 1 hour, but declined thereafter (Fig. S2F and S2G). To further validate the function of transcription factors, we knocked down (KD) *Spi1*, *Rela*, and *Batf* in DCs and measured *Ythdf2* mRNA levels in DCs cocultured with irradiated or un-irradiated tumor cells. We observed that *Spi*1 KD significantly decreased the *Ythdf2* expression in DCs (Fig. 2E), whereas no significant changes in *Ythdf2* expression were observed in *Rela*/*Batf* KD DCs (Fig. S2H, S2I, and S2J). The results suggested that SPI1 is an important transcription factor directly promoting the expression of *Ythdf2*, whereas RELA and BATF transiently bind but not essential for YTHDF2 induction. As SPI1 affects *Ythdf2* expression regardless of IR, we sought to study whether IR induce SPI1 expression. We performed SPI1 Western blots using DCs isolated from tumors 5 days post-IR. The results showed that IR directly increased the SPI1 protein level in a time-dependent manner, with the largest increase at day 3 and 5 post IR (Fig. 2F and S2K). Taken together, these results showed that IR upregulates *Ythdf2* expression by enhancing the protein level of SPI1, which directly binds to the *Ythdf2* promoter and increases *Ythdf2* transcription.

### YTHDF2 loss in CD11c^+^ cells enhances local tumor control and inhibits distant metastasis in radiotherapy

**Figure 3.**
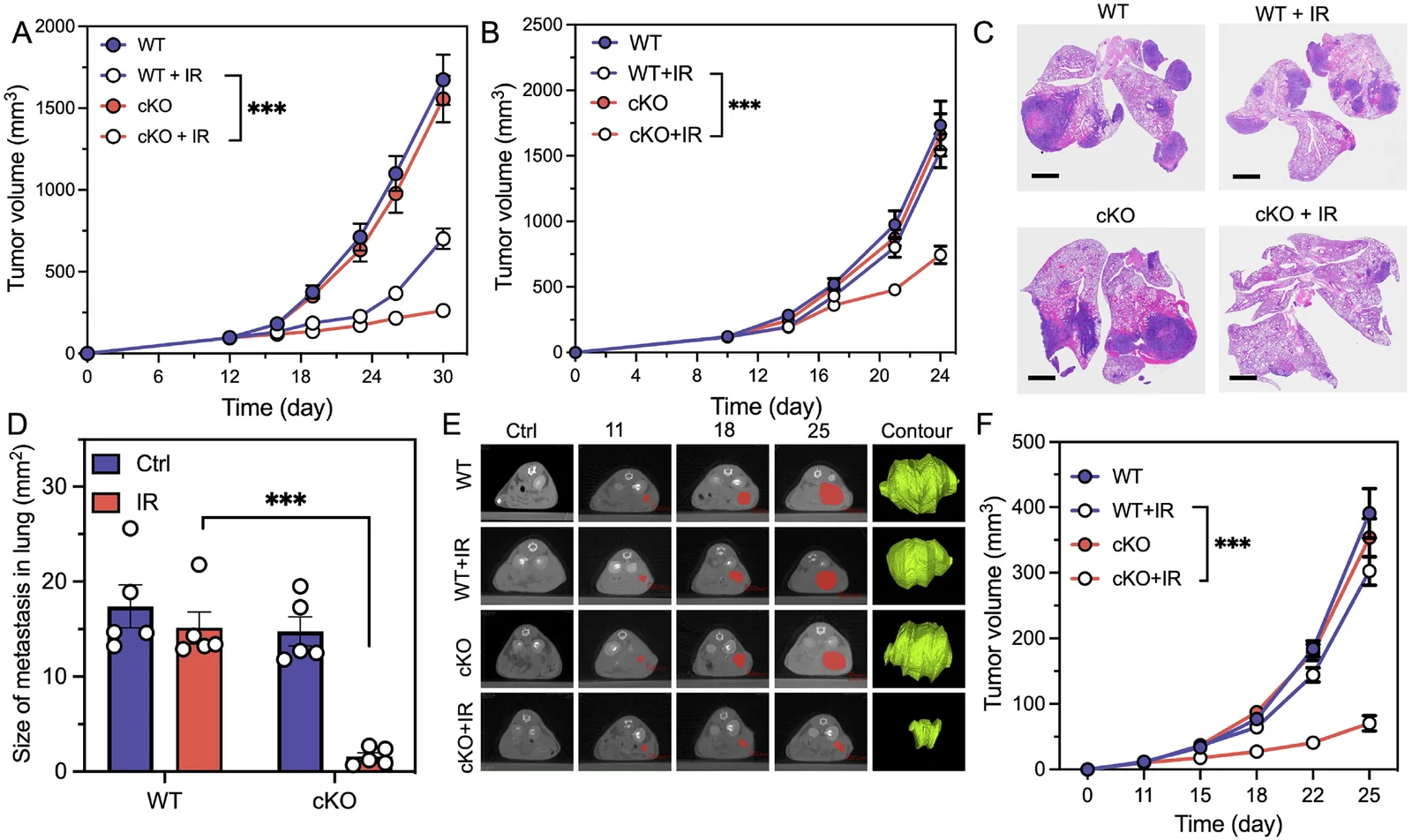
YTHDF2 deficiency in DCs potentiates radiotherapy. (**A**) MC38 tumor growth curves in WT mice or *Ythdf2*-cKO mice with or without IR treatment (20 Gy). 1 million tumor cells were injected subcutaneously (s.c.) and tumors were irradiated when sizes reached around 120 mm^3^ (on day 12) (n = 5, mean ± SD, SD = standard derivation). (**B**) LLC tumor growth curves in WT or *Ythdf2*-cKO mice with or without IR treatment (20 Gy). 1 million tumor cells were injected s.c. and tumors were irradiated when sizes reached around 100 mm^3^ (day 10 post implantation) (n = 5, mean ± SD). (**C**) Hematoxylin and eosin (H&E) staining of lungs of LLC-tumor bearing mice 30 days post tumor inoculation, scale bars: 2 mm. Treatments are as indicated in (B). (**D**) Quantification on total area of metastasis in lungs of (C), using Qupath. (n = 5, mean ± SEM). (**E**) CT images of mice during at different time points (red color indicating tumor area) and representative calculated tumor contours (green) on day 25. KPC tumor cells were injected orthotopically in pancreas. IR treatment (6 Gy dose) was delivered guided by CT imaging on day 11. (**F**) Growth curves of orthotopic KPC tumors in WT or *Ythdf2*-cKO mice with or without IR treatment (n = 5, mean ± SD). Statistical analysis was performed using two-sided unpaired Student’s t test (A, B, D, F); ***P<0.001.

Given that the expression of YTHDF2 in DCs is induced by IR and that the level of YTHDF2 induction was significant in patients who failed to respond to IR and ICI treatment, we hypothesized that YTHDF2 in DCs plays a potential checkpoint role in the context of antigen presentation in the response to radiotherapy. To investigate this hypothesis, we crossed C57BL/6J background *Ythdf2*^flox/flox^ mice with *Itgax*cre (*Cd11c*^Cre^) mice to obtain *Cd11c*^Cre^ *Ythdf2*^flox/flox^ conditional knockout mice (abbreviated as *Ythdf2*-cKO or cKO). *Ythdf2*^f/f^ mice served as wild-type (WT) control for *Ythdf2*-cKO studies. In the MC38 murine colon carcinoma model primary tumor growth of *Ythdf2*-cKO mice was similar with that of WT mice in the absence of IR (Fig. 3A). However, tumor irradiation significantly inhibited the growth of MC38 tumors in *Ythdf2*-cKO mice in comparison with tumors in WT mice. In the B16F10-OT1-zsGreen (B16F10-OZ) melanoma tumor model, we observed a similar phenotype of tumor growth (Fig. S3A) in which B16F10-OZ tumors were significantly inhibited by IR in *Ythdf2*-cKO compared to tumors in WT mice. In addition to improved local tumor response, the survival of tumor-bearing *Ythdf2*-cKO mice was also significantly prolonged following IR compared with WT mice (Fig. S3B).

The enhanced effect of IR in controlling local tumor growth was also observed in Lewis lung carcinoma (LLC) tumors grown in *Ythdf2*-cKO mice compared to tumors in WT mice (Fig. 3B). LLC flank tumors spontaneously metastasize to the lung within 30 days post implantation. Quantification of LLC lung metastases at the study end point showed a reduction in metastatic burden in the lungs of *Ythdf2*-cKO mice of which primary tumors received IR compared with those of WT mice that received IR treatment (Fig. 3C and 3D). To examine whether the *Ythdf2* deficiency in CD11c^+^ cells elicited an anti-tumor effect in an orthotopic model, we used pancreatic cancer cell line derived from KrasLSL-G12D;p53LoxP;Pdx1-CreER mice (KPC344) to establish pancreatic tumors (Fig. S3C). Tumors were imaged by small animal computed tomography (CT) and irradiated using CT-guided RT (Fig. S3C). IR alone did not significantly reduce the pancreatic tumor burden in WT mice; however, IR significantly reduced KPC tumor burden in *Ythdf2*-cKO mice (Fig. 3E and 3F). Taken together, these results demonstrated that the deletion of *Ythdf2* in CD11c^+^ cells significantly enhanced the therapeutic efficacy of radiotherapy not only by improving tumor control of flank or orthotopic primary tumors, but also by inhibiting spontaneous metastases.

### YTHDF2 regulates cross-presentation capacity of DCs in the context of IR

**Figure 4.**
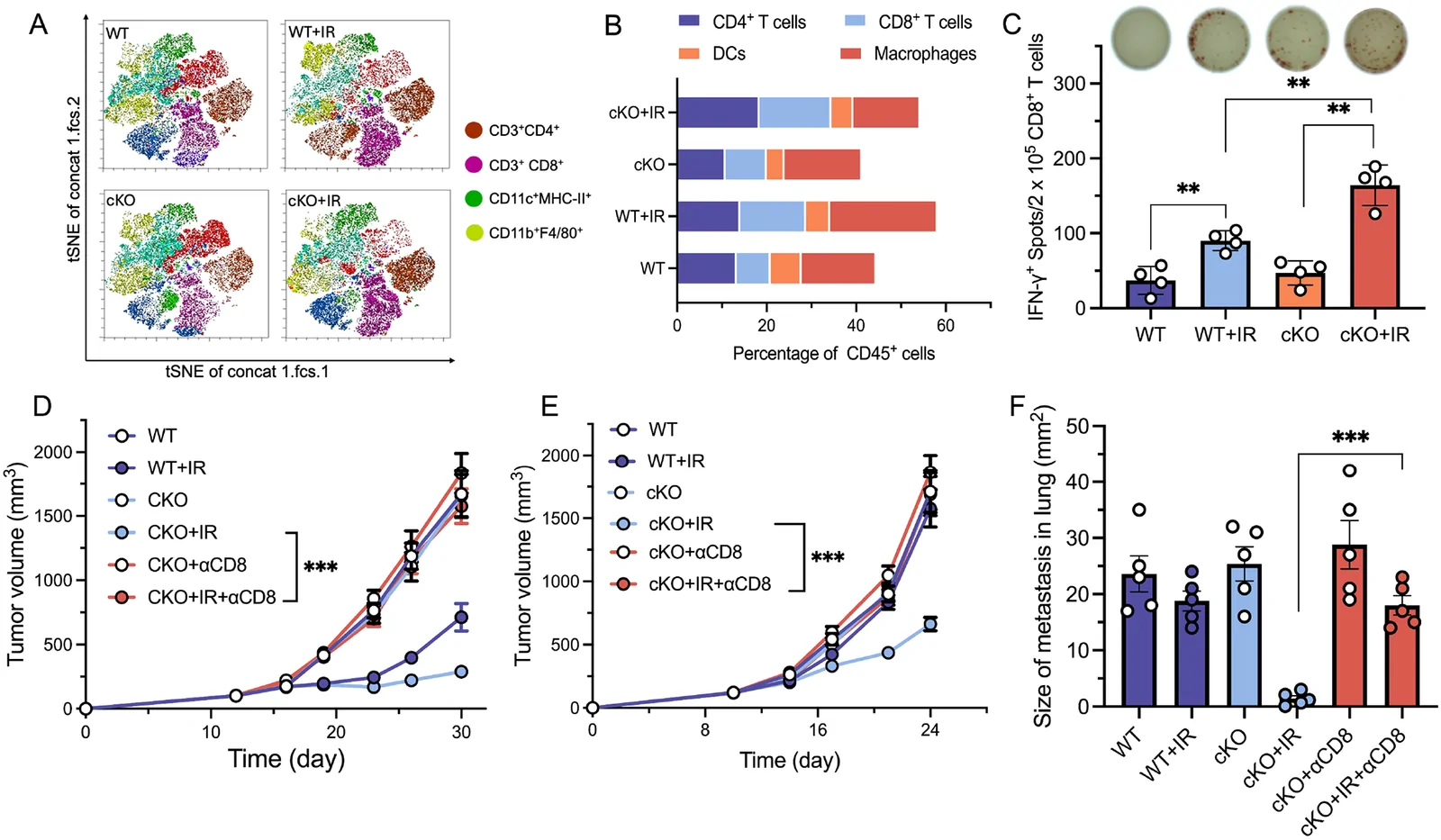
YTHDF2 regulates cross-presentation capacity of DCs in the context of IR. (**A**) tSNE map of different clusters of tumor-infiltrating CD45^+^ immune cells in B16F10 tumors as detected by flow cytometry. (**B**) Populations of infiltrating immune cells in B16F10 tumors assessed by flow cytometry. Macrophages: CD45^+^CD11b^+^F4/80^+^; DCs: CD45^+^CD11c^+^MHC-II^+^Ly6c^−^F4/80^−^; CD8^+^ T: CD45^+^CD3^+^CD8a^+^; CD4^+^ T: CD45^+^CD3^+^CD4^+^. (**C**) ELISPOT assay of IFN-γ positive spots secreted by CD8^+^ T cell. WT, *Ythdf2*-cKO: CD11c^+^ BMDC co-cultured with control B16F10-OZ tumor cells; WT+IR, *Ythdf2*-cKO+IR: CD11c^+^ BMDC co-cultured with irradiated B16F10-OZ tumor cells. (n = 4, mean ± SEM). (**D**) MC38 tumor growth in WT or *Ythdf2*-cKO with or without IR when CD8^+^ T cells were depleted. anti-CD8 antibody were given 200 *μ*g/mouse, twice weekly, starting the day before IR (n = 5, mean ± SD). (**E**) LLC tumor growth in WT or *Ythdf2*-cKO with or without IR when CD8^+^ T cells were depleted. *α*CD8 was administrated at 200 *μ*g/mouse, twice weekly, starting the day before IR (n = 5, mean ± SD). (**F**) Size of LLC spontaneous metastasis total area at the end of the study in (E) and representative images are shown in Figure S3V (n = 5, mean ± SEM). Statistical analysis was performed using two-sided unpaired Student’s t test (C-F); **P<0.01; ***P<0.001.

To examine the impact of *Ythdf2* deficiency in DCs on the tumor immune landscape, we performed spectral flow cytometry analysis of B16 melanoma-infiltrating immune cells. A t-distributed Stochastic Neighbor Embedding (tSNE) clustering analysis (Fig. 4A) demonstrated that IR increased the proportions of CD8^+^ T cells and macrophages in CD45^+^ cells of WT mice (Fig. 4A and 4B), while frequencies of DCs and CD4^+^ T cells were not significantly altered. In tumors grown in *Ythdf2* cKO mice, IR increased the proportions of both CD4^+^ and CD8^+^ T cells in CD45^+^ cells. However, compared to control or irradiated tumors in WT mice, immune cells such as T cells, DCs, and macrophages were not differentially altered in control or irradiated tumors from *Ythdf2* cKO mice, respectively (Fig. S3D–S3I), indicating that the deficiency of YTHDF2 has no significant effect on the composition of tumor-infiltrating immune cells. Given the role of YTHDF2 in DC migration(Liu et al., 2019), we administered 5-(and-6)-carboxyfluorescein diacetate succinimidyl ester (CFSE) labeled BMDCs in B16F10 tumor bearing mice to study whether YTHDF2 affect DC migration in the context of IR. While IR increased the presence of CFSE-labeled DCs in both WT and cKO models (Fig. S3J), the loss of YTHDF2 didn’t significantly increase the presence of CSFE-labeled DCs in comparison with WT models, suggesting that YTHDF2 does not affect the migration capability of DCs. We found no significant differences in CD80 or CD86 expression between the WT and cKO groups in the context of IR (Fig. S3K and S3L), suggesting that loss of YTHDF2 does not influence canonical DC maturation under our experimental conditions. Moreover, cell viability was comparable between WT and cKO DCs pre- or post- coculture with CD8^+^ T cells (Fig. S3M). Therefore, the enhanced anti-tumor effect is unlikely to result from altered viability of cKO DCs.

We next examined the effects of *Ythdf2* on antigen presentation by DCs in the context of IR. *In vitro* cross-priming assays utilized BMDCs from WT and *Ythdf2*-cKO mice that were cocultured with control or irradiated B16F10-OZ tumor cells (DCs±IR), and CD11c^+^ DCs were subsequently purified from the mixture and cocultured with CD8^+^ T cells from OT1 mice for interferon-γ (IFN-γ) ELISPOT assays. As indicated by IFN-γ production by CD8^+^ T cells, CD8^+^ T cell cross-primed by *Ythdf2*-cKO DCs+IR was significantly augmented compared with WT DCs+IR (Fig. 4C). YTHDF2 inhibitor DC-Y13–27 treated DCs+IR also showed enhanced cross-priming activity for CD8^+^ T cells compared with WT DCs+IR (Fig. S3N) similar with the activity observed in *Ythdf2*-cKO BMDCs+IR (Fig. S3O), confirming a central role of YTHDF2 in regulating cross-priming activity of DCs in radiotherapy. We further assessed the antigen cross-presentation of DCs using B16-OVA tumor model, which express full length ovalbumin. We detected a significant increase of H2Kb-SIINFEKL density in CCR7^+^ DCs migrated from B16-OVA tumors inoculated cKO mice in comparison with WT mice (Fig. S3P). In functional assays, the cKO DCs induced enhanced IFN-γ production from OT-I CD8+ T cells (Fig. S3Q), confirming that the loss of YTHDF2 enhances the antigen cross-presentation of DCs. To determine whether YTHDF2 regulate direct peptide loading by MHC-I, we performed ELISPOT assay on IFN-γ^+^ CD8^+^ T cells cocultured with WT or cKO DCs pulsed with SIINFEKL. Across addition of different doses of SIINFEKL, we observed comparable IFN-γ secretion between co-cultures of WT and cKO DCs, suggesting that YTHDF2 loss will not affect the direct antigen loading by MHC-I (Fig. S3R). To further assess whether cross-dressing is regulated by YTHDF2, we co-cultured irradiated 4T1-HA tumor cells (H2Kd background) with DCs isolated from WT and cKO mice (H2Kb background). There was no significant change on the surface expression of H2Kd on DCs of cKO compared to WT DCs after co-culture with 4T1-HA cells (Fig. S3S), suggesting that *Ythdf2*-deficiency will not affect the acquisition of exogenous MHC-I (H2Kd). Next, we co-cultured these DCs with CD8^+^ T cells and analyzed antigen-specific responses using H2Kd-HA tetramer staining. We did not observe a significant difference in tetramer^+^ CD8^+^ T cells between the WT and cKO groups (Fig. S3T), indicating that *Ythdf2* deletion does not significantly enhance cross-dressing of DCs with exogenous antigens.

To determine whether CD8^+^ T cells are required for the enhanced response to IR in Yth*df2*-cKO mice, we depleted CD8^+^ T cells using a neutralizing antibody (αCD8) in MC38 and B16F10-OZ tumors. We observed that CD8^+^ T cell depletion completely abrogated the enhanced anti-tumor effects of IR in *Ythdf2*-cKO mice (Fig. 4D and S3U). The depletion of CD8^+^ T cells also abolished the superior control of LLC local tumors (Fig. 4E) and metastases (Fig. 4F and S3V) by IR in *Ythdf2*-cKO, indicating that CD8^+^ T cells are essential for the enhanced control of local and distant disease in irradiated *Ythdf2*-cKO mice. Taken together, these findings suggest that loss of YTHDF2 potentiates the cross-presentation capacity of DCs in radiotherapy, thus leading to superior activation of CD8^+^ T cells and ultimate systemic disease control.

### IR-induced YTHDF2 degrades m^6^A-bound mRNA in Notch signaling pathway

**Figure 5.**
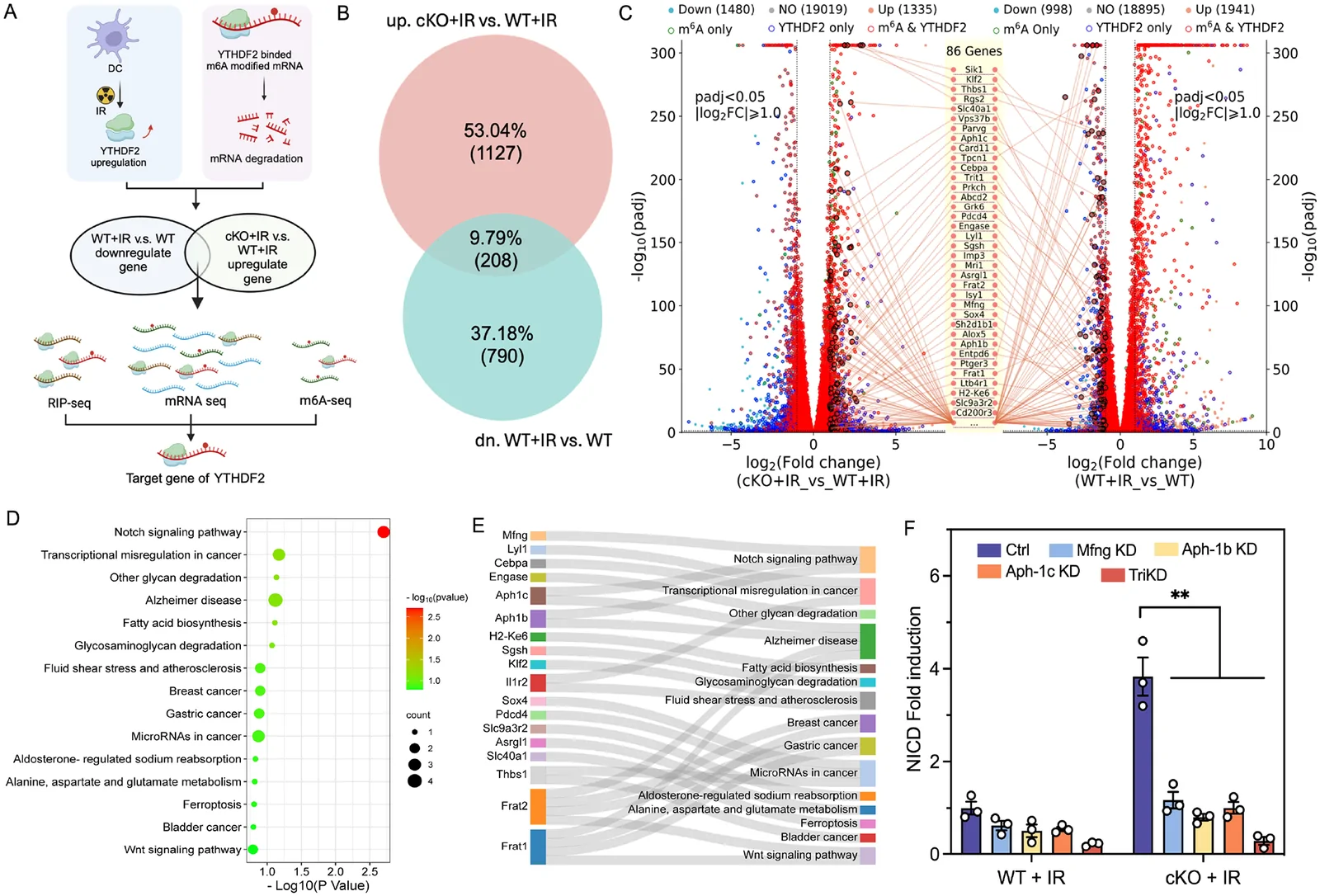
IR induces YTHDF2 to degrades m6A-bound mRNA in Notch signaling pathway. (**A**) Schematic illustration on the analysis strategy for identifying YTHDF2-bound and m^6^A-modified gene transcripts. (**B**) Venn diagram of overlapping genes from mRNAseq that were downregulated following WT+IR versus WT and upregulated following *Ythdf2*-cKO+IR versus WT+IR. (**C**) Twin-volcano plot of genes with differential expression levels in the DCs (left: cKO+IR versus WT+IR, right: WT+IR versus WT), m^6^A marked genes (m^6^A-seq) are shown with green circles, YTHDF2 marked genes (RIPseq) are shown with purple circles, both m6A and YTHDF2 marked genes are shown with red circles. 86 target genes of YTHDF2 shown in upregulated cKO+IR versus WT+IR region and downregulated WT+IR versus WT region are represented with black circles. (**D**) GO enrichment pathway analysis using 86 YTHDF2 target genes in the context of IR. (**E**) Sankey flow diagrams of specific genes categorized in each enriched pathway in (D). (**F**) Induction of NICD from BMDCs with single or triple knockdown of *Mfng*, *Aph-1b*, and *Aph-1c*, n = 3. Statistical analysis was performed using two-sided unpaired Student’s t test (F); **P<0.01. Data are represented as mean ± SEM.

To explore the mechanisms by which YTHDF2 regulates DC function in the context of IR, we analyzed the RNA-seq of tumor-infiltrating DCs from WT and *Ythdf2*-cKO mice treated with or without radiation. Since an important function of YTHDF2 is in degradation of m^6^A-modified RNA, we proposed the target of YTHDF2 is shared between the down-regulated genes in WT+IR vs WT and the up-regulated genes in cKO+IR versus WT+IR (Fig. 5A). Comparing WT+IR versus WT, we found that radiation leads to down-regulation of 998 genes and upregulation of 1941 genes (Fig. 5B, S4A and S4B). Comparing cKO+IR versus WT+IR, we found that the loss of YTHDF2 leads to 1335 upregulated genes and 1480 downregulated genes in DCs post-IR (Fig. 5B, S4A and S4B). There were 537 genes are shared between downregulated genes of cKO+IR versus WT+IR and upregulated genes in WT+IR versus WT (Fig. S4A) and 208 genes are shared between upregulated genes of cKO+IR versus WT+IR and downregulated genes in WT+IR versus WT (Fig. 5B).

To further identify direct targets that both have m^6^A modifications and YTHDF2-binding (Fig. 5A), we performed YTHDF2-RNA immuno-precipitation sequencing (RIPseq) and m^6^A RNA immuno-precipitation sequencing (MeRIP-seq) to identify the YTHDF2-bound RNAs and reveal m^6^A-modified mRNAs, respectively. Following this strategy, we found 86 genes (e.g. *Sik1*, *Klf2*, *Thbs1*) as targets of YTHDF2 and with m^6^A modification in the context of IR (Fig. 5C). GO enrichment analysis of these genes indicated that the Notch signaling pathway was among the top enriched pathways (Fig. 5D). Specifically, three target genes (*Mfng*, *Aph1b*, and *Aph1c*) are known positive regulators of the Notch signaling pathway (Fig. 5E). *Mfng* is known to promote elongation on O-linked fucose residues on Notch 1 receptors.(Kakuda and Haltiwanger, 2017) *Aph1b* and *Aph1c* are important in the composition of γ-secretase, which plays a significant role in the cleavage of Notch intracellular domains (Fig. S4C).(Serneels et al., 2005; Shirotani et al., 2004) We confirmed the direct YTHDF2 binding by immunoprecipitation (IP) using YTHDF2 antibody for mRNA pull-down and found that YTHDF2 directly binds to all three transcripts (Fig. S4D).

To investigate the molecular interactions between YTHDF2 and the Notch pathway target genes (*Mfng*, *Aph1b*, and *Aph1c*), we studied the half-life of these transcripts with and without the presence of YTHDF2 and found that increased half-lives of these transcripts in *Ythdf2*-cKO DCs compared to WT DCs (Fig. S4E–S4G), consistent with the known function of YTHDF2 in promoting mRNA degradation. In tumor-infiltrating DCs, we observed decreased mRNA levels of *Mfng*, *Aph1b*, and *Aph1c* in DCs from irradiated tumors in WT mice compared with DCs from non-irradiated WT mice. Conversely, expression of these genes was increased in DCs of *Ythdf2*-cKO compared with that of DCs from WT mice in the context of IR. The data suggested that YTHDF2 negatively regulates Notch signaling by reducing the expression of *Mfng*, *Aph1b*, and *Aph1c* following IR (Fig. S4H). To determine which Notch ligands and receptors are involved, we performed qPCR to profile Notch receptors (Notch1, Notch2, Notch3, Notch4) on dendritic cells and Notch ligands (Jagged1, Jagged2, DLL1) on tumor cells in our co-culture system. Among these, we found that Notch1 on DCs were significantly upregulated in the context of YTHDF2 deficiency (Fig. S4I). Moreover, the ligand DLL1 on tumor cells were significantly upregulated in cells receiving irradiation (shown Fig. S4J). These results suggest that the Notch1–DLL1 axis is the most relevant ligand–receptor interaction mediating the enhanced cross-priming observed in our study. This finding aligns with the proposed mechanism in which YTHDF2 modulates DC function through regulation of Notch signaling components.

To characterize the functions of these YTHDF2-regulated genes, we knocked down (KD) genes individually or together in an engineered Notch 1 intracellular domain (NICD) reporter system in Flt3l DCs and co-cultured the DCs with control or irradiated tumor cells. No significant reduction of NICD was observed in WT DCs with any single gene KD during co-culture with irradiated tumor cells. *Ythdf2*-cKO DCs co-cultured with irradiated tumor cells had significantly induced NICD compared to WT DCs. Single KD or TriKD of the target genes resulted in significant decrease of the NICD induction compared to *Ythdf2*-cKO scramble control (Fig. 5F). Collectively, these results suggested that YTHDF2 negatively regulate the Notch signaling pathway by targeting and degrading the relevant genes (*Mfng*, *Aph1b*, and *Aph1c*) in the context of IR.

### The loss of YTHDF2 in DCs induces expression of MHC-I gene *Gm8909*

**Figure 6.**
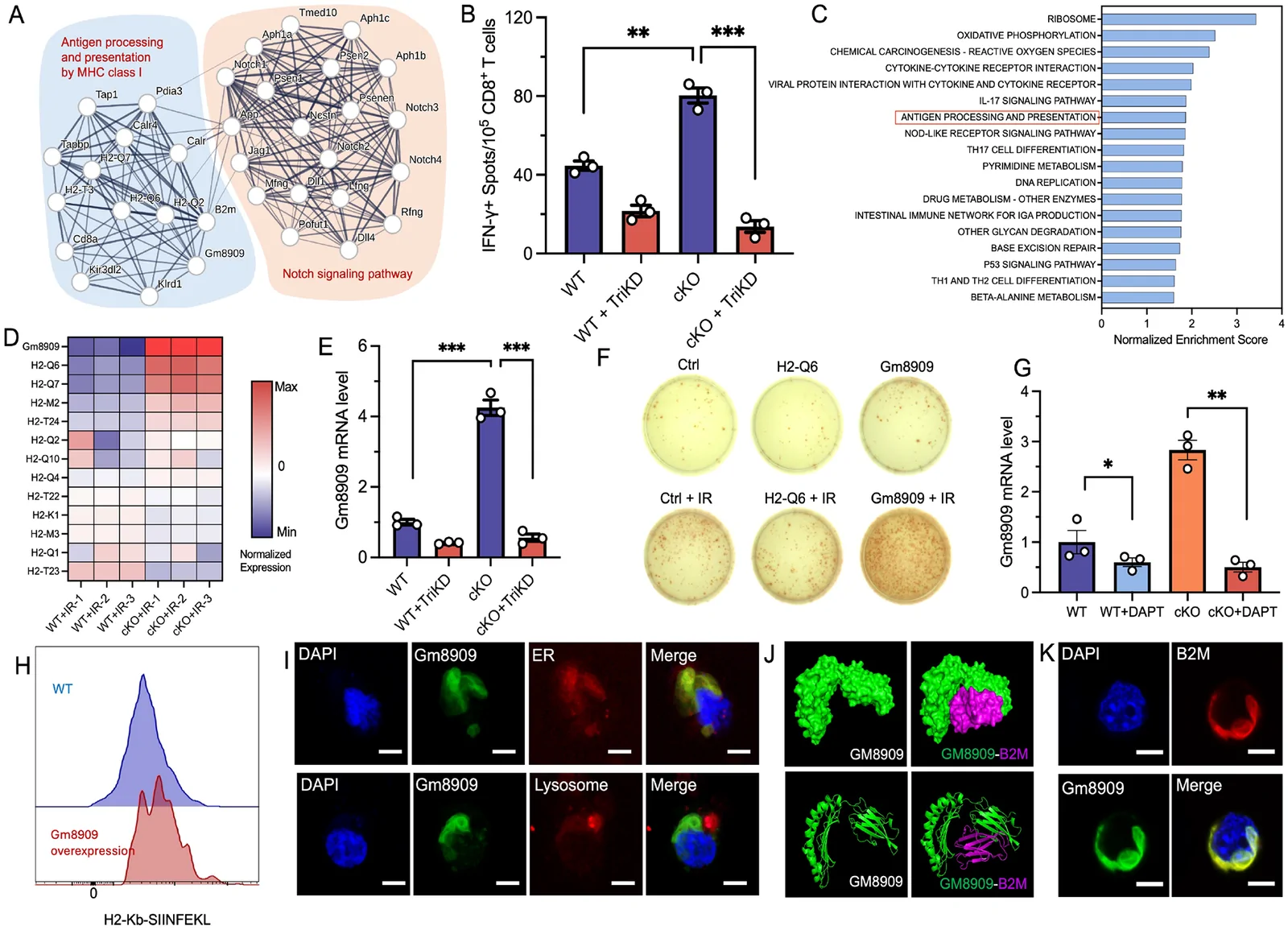
The loss of YTHDF2 in DCs induces Gm8909 encoded MHC-I by Notch signaling pathway. (**A**) STRING protein-protein interaction network between the MHC-I pathway (involving Gm8909 and H2-Q6) and Notch signaling pathways (involving *Mfng*, *Aph-1b*, and *Aph-1c*). The minimum required interaction score was set to 0.4. k-means clustering was applied. Line thickness denoted the STRING PPI score/confidence. (**B**) ELISPOT assay on IFN-*γ* secreted by CD8^+^ T cells co-cultured with WT/TriKD DCs which were previously exposure to irradiated B16F10-OZ cells (n = 3). (**C**) GSEA pathway analysis indicating antigen processing and presentation pathway enriched in DCs (cKO+IR versus WT+IR). (**D**) Heatmap showing the expression of *Gm8909*, *H2-Q6* and other MHC-I relevant genes in tumor-infiltrating DCs from WT and *Ythdf2* cKO mice. (**E**) *Gm8909* mRNA expression in WT or *Ythdf2*-cKO DCs which were cocultured with irradiated tumor cells. TriKD indicates DCs knockdown with *Mfng*, *Aph-1b* and *Aph-1c* siRNA (n = 3). (**F**) ELISPOT assay on IFN-*γ* secreted by CD8^+^ T cells in coculture with H2-Q6/GM8909 overexpressed DCs, which were previously treated with DAPT and exposure to irradiated B16F10-OZ cells. (**G**) mRNA expression of *Gm8909* in WT or cKO BMDCs with or without treatment of inhibitor DAPT, n = 3. (**H**) H2-Kb-SIINFEKL level in WT DCs or DCs overexpressed with GM8909. (**I**) Confocal fluorescence imaging of DCs for detecting subcellular localization of GM8909 in ER or lysosomes, scale bars: 5 μm. (**J**) Structure of GM8909 and ClusPro protein-protein docking between GM8909 and B2M. (**K**) Confocal fluorescence imaging of DCs for detecting subcellular localization GM8909 with B2M, scale bars: 5 μm. Statistical analysis was performed using two-sided unpaired Student’s t test (B, E, G), *P<0.05, **P<0.01, ***P<0.001. Data are represented as mean ± SEM.

Given that YTHDF2 directly targets the Notch signaling pathway *via* decreasing relevant transcripts of signaling components (*Mfng*, *Aph1b*, and *Aph1c*) and loss of YTHDF2 enhances antigen cross-presentation function of DCs, we performed network analysis to investigate potential interactions between Notch signaling and MHC-I pathways (Fig. 6A). A STRING protein-protein interaction (PPI) network(Szklarczyk et al., 2023) analysis showed that there are multiple potential interactions (e.g. CALR-APP, B2M-NOTCH1, PDIA3-APH1A) between these two pathways (Fig. 6A), suggesting that the YTHDF2-targeted Notch signaling pathway might impact the MHC-I activity in DCs following irradiation. To test this, we examined the antigen cross-presentation capacity of BMDCs following knock down of *Mfng*, *Aph1b*, and *Aph1c* (triKD). We observed that upon co-culturing with irradiated tumor cells, triKD of these transcripts in both WT and *Ythdf2*-cKO DCs led to reduced IFN-γ secretion by CD8^+^ T cells (Fig. 6B, S5A, S5B, S5C, and S5D), suggesting the Notch pathway is important to DC cross-presentation function.

To investigate the molecular mechanism of antigen cross-presentation enhanced by YTHDF2 deficiency, we conducted gene set enrichment analysis (GSEA) on RNA-seq data in DCs from cKO+IR versus WT+IR (Fig. 6C). We observed that the antigen presentation pathway was significantly upregulated in cKO+IR DCs, represented by genes such as *Cd8a*, *Gm8909*, *H2-Oa* and *H2-Q6* (Fig. S5E). Among them, the expression of *Gm8909* and *H2-Q6*, were significantly elevated in DCs from cKO+IR compared with WT+IR (Fig. S5E and 6D). Both *Gm8909* and *H2-Q6* belong to MHC-I family genes, predicted with MHC antigen recognition domain and functions in antigen peptide loading, processing, and presentation to T cell receptor (TCR).(Perez et al., 2024) To validate this finding, we collected tumor-infiltrating DCs and quantified the expression level of *Gm8909* and *H2-Q6* transcripts with qPCR. The expression of both transcripts was significantly upregulated in *Ythdf2*-cKO DCs from irradiated tumors (Fig. S5F and S5G), confirming that *Gm8909* and *H2-Q6* are regulated by YTHDF2 in the context of IR.

To characterize the relationship between the Notch signaling pathway and MHC-I gene expression, we measured the expression of MHC-I genes *Gm8909* and *H2-Q6* in BMDCs following triKD of the YTHDF2 targets in the Notch pathway (*Mfng*, *Aph1b*, and *Aph1c*). The result showed that expression of both *Gm8909* and *H2-Q6* were significantly downregulated with triKD (Fig. 6E and S5H). In DCs treated with the Notch pathway inhibitor DAPT, the expression of *Gm8909* and *H2-Q6* at mRNA level also decreased. These results indicate that inhibition of Notch signaling pathway negatively regulates the transcription of MHC-I genes (Fig. 6G and S5I). To determine the function of MHC-I genes *Gm8909* and *H2-Q6* in DCs upon IR, *H2-Q6* and *Gm8909* were overexpressed in BMDCs and co-cultured with irradiated or control (un-irradiated) tumor cells for antigen cross-presentation assays. As indicated by the IFN-γ spots secreted by CD8^+^ T cells when cocultured with irradiated tumor cells, *Gm8909*-overexpressed (*Gm8909*^+^) DCs showed significantly enhanced cross-presentation function compared to those of control DCs or *Gm8909*^+^ DCs co-cultured with un-irradiated tumors (Fig. 6F and S5J). A similar effect was not observed in *H2-Q6* over-expressed DCs. To investigate the impact of Notch signaling pathway to MHC-I function, we treated *Gm8909* and *H2-Q6* overexpressed DCs using inhibitor DAPT. While DAPT-treated control DCs showed decreased cross-priming capacity, cross-priming capacity of DAPT treated *Gm8909*^+^ DCs remained significantly increased compared with DAPT alone or DAPT+*H2-Q6*^+^ group (Fig. S5K and S5L). We also observed that after co-culture with irradiated B16F10-OZ cells, DCs overexpressing *Gm8909* showed a higher level of H2-Kb-SIINFEKL than WT DCs (Fig. 6H) suggesting that *Gm8909* in DCs promotes other MHC-I family protein for antigen cross-presentation. Gm8909 overexpression does not affect the H2-Kb expression in DCs (Fig. S5M), which suggests that Gm8909 selectively enhances antigen presentation efficiency, rather than driving a general upregulation of MHC-I surface levels. We knocked down *Gm8909* using siRNA in cKO BMDCs and co-cultured them with CD8^+^ T cells in the presence of irradiated B16F10-OZ cells. The results showed a significant reduction in IFN-γ–producing CD8^+^ T cells (Fig. S5N), indicating that *Gm8909* is indeed required for the elevated cross-priming activity in cKO DCs. Taken together, these results demonstrates that GM8909 plays an important role in MHC-I mediated antigen cross-presentation of DCs in the context of IR. The Notch signaling pathway, which is targeted by IR induced YTHDF2, plays a significant role in sustaining GM8909 expression and antigen-presentation function in DCs.

GM8909 was predicted to play a role in antigen processing and presentation of endogenous antigen *via* endoplasmic reticulum (ER) pathway.(Perez et al., 2024) To test this, we performed confocal microscopy of *Gm8909*^+^ DCs that were tagged with GFP. The fluorescence of GM8909 colocalized with ER trackers in DCs but to less of an extent with lysosomal trackers (Fig. 6I), consistent with the prediction that GM8909 preferentially localized to the ER. We also observed that in DCs co-cultured with tumor cells, GM8909-GFP translocated to the plasma membrane from cytoplasm of DCs (Fig. S5O), which may facilitate antigen cross-presentation. Beta-2-microglobulin (β2M) is an essential light-chain for assembling with heavy-chain to form MHC-I complex for peptide loading. To detect the affinity of β2M to GM8909, we conducted protein-protein dock calculations with ClusPro (Desta et al., 2020; Jones et al., 2022; Kozakov et al., 2017; Vajda et al., 2017) and observed a high docking energy at −204.9 kcal/mol of Vander Waals. The algorithm also detected electrostatic interactions between β2M chain and GM8909 chain. These predictions suggested a strong binding affinity of β2M to GM8909 chain (Fig. 6J). Immunofluorescent microscope imaging showed that β2M is co-localized with GM8909 in DCs (Fig. 6K). Using a GFP-tagged GM8909 construct, we performed co-IP with an anti-GFP antibody, followed by western blot analysis, which revealed a specific association between GM8909 and β2M (Fig, S5P). This finding suggests that GM8909 may facilitate or stabilize MHC-I complex formation or trafficking through direct interaction with β2M. Taken together, these results suggested that GM8909 may serve as the heavy chain to assemble with β2M to form MHC-I complex for antigen loading and presentation.

### Targeting YTHDF2 promotes efficacy of DC vaccines to enhance radiotherapy

*Ex vivo*-generated, antigen-loaded DCs have been used as adjuvants for vaccination in patients with cancer, eliciting safe, therapeutic, and protective antitumor immunity.(Palucka and Banchereau, 2013; Timmerman and Levy, 1999) Despite this, clinical responses of DC vaccines remain low with objective response rates not exceeding 15%,(Anguille et al., 2014) prompting efforts to refine immunological and clinical parameters to improve its efficacy. Given that *Ythdf2*-deficient DCs exert superior cross-priming capacity, we sought to generate prototype DC vaccines to enhance the anti-tumor immunity of radiotherapy. To test the efficacy of a prototype DC vaccine, we injected WT or YTHDF2-deficient Flt3l DCs into B16F10-OVA tumors 3 times/week. Compared to control groups, WT DC injection significantly decreased tumor growth (Fig. S5Q). Further, cKO DC vaccines significantly enhanced anti-tumor effects compared with WT DC vaccines. In addition, pre-treatment of WT DCs with YTHDF2 inhibitor DC-Y13–27 augmented anti-tumor effects, consistent with the results of *Ythdf2* cKO DCs (Fig. S5R). The results showed that DC vaccines with YTHDF2 modulation indeed increased anti-tumor effects.

**Figure 7.**
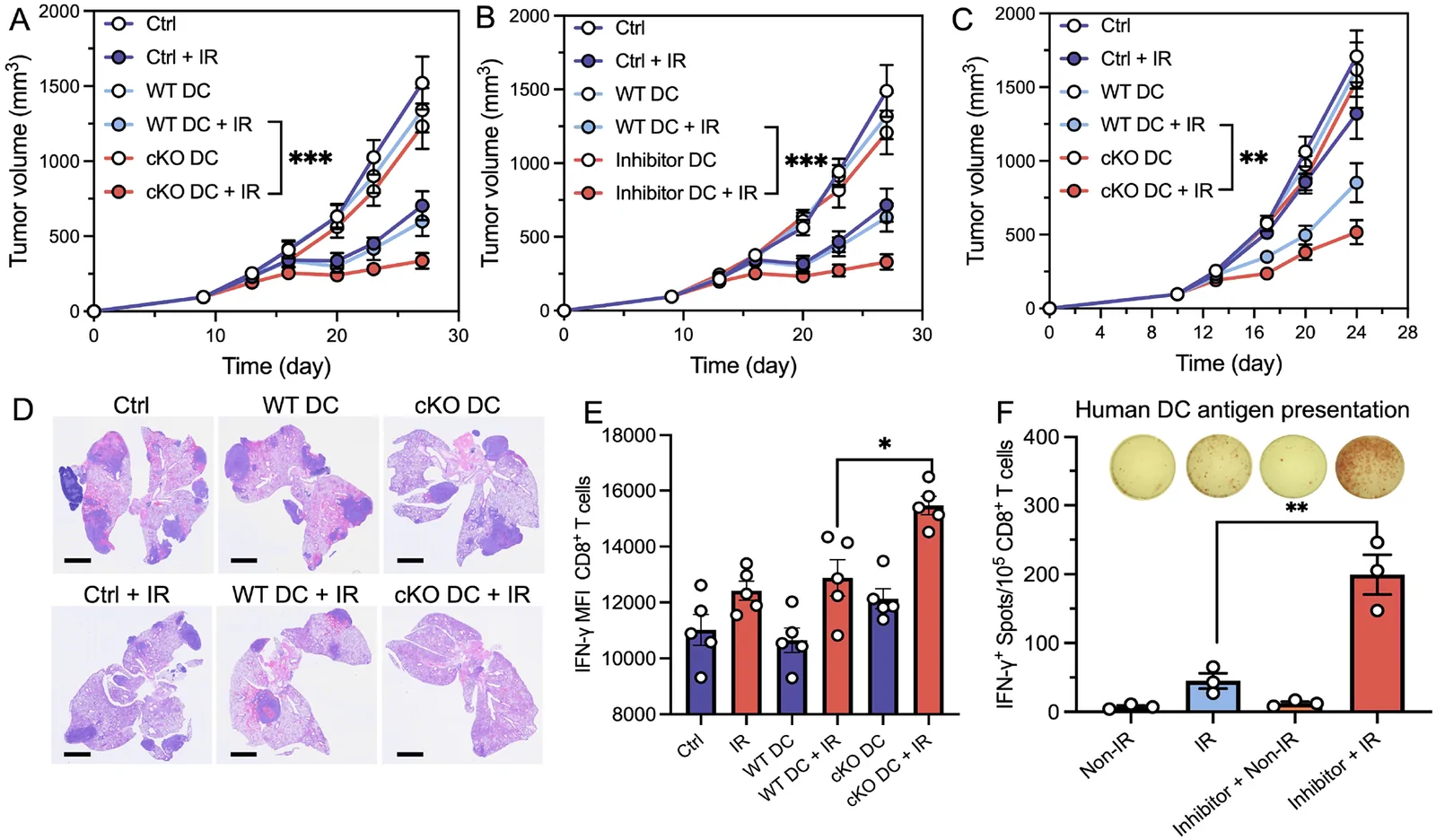
YTHDF2 inhibition promotes DC vaccines efficacy with radiotherapy. (**A**) B16F10-OVA tumor growth curves with indicated treatments. WT and *Ythdf2*-cKO DC vaccines were administered intratumorally once per week. Tumors were treated with or without IR (20 Gy) on day 9 (n = 5, mean ± SD). **(B**) B16F10-OVA tumor growth curves with indicated treatments. WT DCs or WT DCs treated with YTHDF2 inhibitor were administered intratumorally once per week. Tumors were treated with or without IR (20 Gy) on day 9 (n = 5, mean ± SD). (**C**) LLC tumor growth curves with indicated treatments. WT DCs or *Ythdf2*-cKO DC were administered intratumorally once per week. Tumors were treated with or without IR (20 Gy) on day 9 (n = 5, mean ± SD). (**D**) Representative LLC spontaneous lung metastasis of mice receiving treatments as indicated in (C), scale bars: 2 mm. (**E**) IFN-γ^+^ CD8^+^ T cells in lung tissue. Lung was collected on day 10 post IR treatment (n = 4, mean ± SEM). (**F**) ELISPOT assay of human DC to assess antigen-cross presentation capability to matched human T cells. DCs were treated with YTHDF2 inhibitor and further co-cultured with irradiated or non-irradiated Hct116 cells (n = 5, mean ± SEM). Statistical analysis was performed using two-sided unpaired Student’s t test (A- C, E-F); *P<0.05; **P<0.01; ***P <0.001.

To test whether DC vaccine with YTHDF2 inhibition affects the efficacy of radiotherapy, we administered WT or YTHDF2-deficient Flt3l DCs at a lower frequency (1 time/week) into B16F10-OVA tumor-bearing mice, starting on the day of radiation. WT DCs exhibited no significant effect on the growth of B16F10-OVA tumors in comparison with control groups. Tumors treated with cKO DC vaccine alone showed similar growth to the tumors treated with WT DC vaccine. Combination of radiation with cKO DC vaccine resulted in the greatest decrease in B16F10-OVA tumor growth when compared with WT DC+IR treatment (Fig. 7A). Similarly, DCs pre-treated with YTHDF2 inhibitor DC-Y13–27 transferred at 1 time/week did not show significant effects alone. However, consistent with cKO DCs results, DCs treated with the inhibitor significantly improved anti-tumor effect of radiation compared to either single treatment (Fig. 7B).

We tested low dose (1 dose/week) DC vaccine in the LLC spontaneous lung metastasis model. WT DC vaccines exhibited no significant effect on the growth of LLC flank tumors in comparison with control groups. cKO DC vaccine alone showed similar effects on tumor growth as the WT DC vaccine. The combination of radiotherapy with cKO DC vaccine resulted in a significant inhibition of LLC tumor growth when compared with WT DC vaccine + IR therapy (Fig. 7C). Again, the effect of YTHDF2 inhibitor DC-Y13–27 pre-treated DCs showed a similar phenotype as cKO DCs in the LLC tumor model (Fig. S5S). A reduced burden of spontaneous LLC lung metastases was observed in lungs of YTHDF2-inhibitor treated DC vaccine group that received IR compared with WT DC group that received IR (Fig. S5T and S5U). We also observed similarly reduced metastatic area in the lungs of *Ythdf2*-cKO DC vaccines + IR group in comparison with WT DC vaccine + IR group (Fig. 7D and S5V). To determine whether the activity of the YTHDF2 inhibitor DC-Y13–27 depends on YTHDF2 expression in dendritic cells, we treated cKO DCs with the inhibitor and found no further enhancement in tumor growth control compared to cKO DCs alone (Fig. S5W), suggesting that the inhibitor’s effects are YTHDF2-dependent. To examine the global impact of *Ythdf2* deficiency on the lung immune microenvironment, we conducted flow cytometry of immune cells from lungs of mice inoculated with LLC cells 10 days post the start of treatments. We observed that in the mice treated with *Ythdf2*-cKO DC vaccine, IR increased the level of IFN-γ in CD8^+^ T cells compared to that of mice treated with WT DC vaccines (Fig. 7E), indicating that the DCs deficient of YTHDF2 significantly improved the function of CD8^+^ T cells in mouse lung. To assess tumor antigen–specific CD8+ T cell responses, we used LLC-OT-1 spontaneous metastasis model. Tumor-antigen specific H2Kb–SIINFEKL tetramers were used to stain T cells, and a significantly increase of tetramer mean fluorescence intensity was observed in OT-I CD8+ T cells primed with cKO DCs (Fig. S5X), indicating more efficient antigen-specific cross-priming. Taken together, these data demonstrated that prototype DC vaccines in which *Ythdf2* is deleted or inhibited enhanced the efficacy of radiotherapy by increasing local and distant metastasis control.

To investigate YTHDF2’s role in human DC function, we induced human DCs from monocytes derived from PBMCs of an EBV^+^ donor. The induced DCs were co-cultured with CD8^+^ T cells isolated from matched EBV^+^ PBMC for antigen cross-priming assays. We found that consistence with the outcomes of mouse DCs, human DCs co-cultured with irradiated EBV^+^ HCT116 cells subsequently enhanced IFN-γ secretion by CD8^+^ T cells, compared to the control group DCs which were co-cultured with un-irradiated tumor cells. Moreover, after co-culture with irradiated tumor cells, DCs pre-treated with YTHDF2 inhibitor resulted in significantly enhanced IFN-γ secretion by CD8^+^ T cells in comparison with that of control untreated DCs (Fig. 7F), indicating that the inhibition of YTHDF2 in human DCs enhances antigen cross-presentation function.

## Discussion

We demonstrate that YTHDF2, an m^6^A-dependent mRNA degrader, functions as a radiation-induced immune checkpoint in dendritic cells (DCs), suppresses anti-tumor immunity after radiation exposure. Analysis of patient samples and animal models revealed that RT upregulates YTHDF2 in DCs, impairing antigen presentation and promoting immune evasion. Loss of *Ythdf2* in DCs enhanced the local anti-tumor effects of RT and significantly suppressed metastasis development. Using multiple tumor models, including an orthotopic pancreatic tumor model, we found that YTHDF2 depletion or pharmacological inhibition significantly improved RT tumor control and reduced lung metastasis. Notably, the same effect was observed with DC vaccines engineered to block YTHDF2, highlighting its potential as a therapeutic target.

Mechanistically, IR-induced SPI1 directly binds to the *Ythdf2* promoter, increasing its expression in DCs. Elevated YTHDF2 levels promote m^6^A-mediated degradation of *Aph-1b*, *Aph-1c*, and *Mfng*—key regulators of Notch signaling, which lead to impaired MHC-I antigen presentation and reduced CD8^+^ T cell priming. The loss of YTHDF2 restores Notch signaling, which results in upregulation of *Gm8909* (a member of the MHC-I family), and enhances antigen cross-presentation, ultimately improving DC-mediated anti-tumor immunity in response to RT.

Beyond revealing a new function of YTHDF2, our study highlights a key distinction from YTHDF1,(Han et al., 2019; Wen et al., 2024) which primarily regulates antigen processing by promoting lysosomal cathepsin translation. YTHDF2, in contrast, directly suppresses MHC-I antigen presentation by destabilizing Notch pathway transcripts, impairing CD8^+^ T cell activation. While previous studies linked YTHDF2 to immune suppression in MDSCs,(Wang et al., 2023) macrophages,(Ma et al., 2023) and B cells,(Chen et al., 2024) our work demonstrates its role in DCs as a regulator of antigen presentation and introduces a novel, clinically relevant approach—leveraging YTHDF2 inhibition to improve DC-based cancer vaccines. Given the crucial role of DC vaccines in tumor immunotherapy, targeting YTHDF2 offers a promising strategy to enhance RT efficacy and prevent metastasis.

To translate these findings into a therapeutic approach, we developed proof-of-principle DC vaccines treated with a YTHDF2 inhibitor or genetical depletion of *Ythdf2*. Intra-tumoral administration of these modified DC vaccines significantly enhanced the anti-tumor immunity and metastasis-suppressive effects of RT, reinforcing their clinical potential. Despite limited response rates in clinical trials, DC-based cancer vaccines remain a focus in immuno-oncology. Our results suggest that targeting YTHDF2 could significantly improve the efficacy of these vaccines, particularly given the limited effectiveness of current DC vaccines and the need for strategies to enhance their therapeutic potential. The combination of DC vaccination and YTHDF2 inhibition presents a safe, personalized, and clinically viable strategy for next-generation DC immunotherapies.

### Limitations of study

While we identify YTHDF2 as a key regulator of RT-induced immune suppression in DCs, our study has some limitations. Although YTHDF2 upregulation was most pronounced in DCs after RT, its interactions with other immune cells remain unclear, and it may also play a role in other immune-related diseases. This study primarily used CD11c^+^ cells-targeted *Ythdf2* deletion, which may not fully capture its function in specific DC subpopulations. CD11c^+^ expression is not confined to DCs, and that YTHDF2 deletion may affect other CD11c-expressing immune cells within the tumor. Additionally, while YTHDF2 depletion enhanced CD8^+^ T cell priming in the lungs, it is uncertain whether this effect is driven by DC activity or T cell trafficking. Further studies are also needed to determine whether *Aph-1b*, *Aph-1c*, and *Mfng* influence other Notch family members or additional biological pathways. Finally, the effects of YTHDF2 inhibition in human DCs should be carefully assessed, considering genetic and immune heterogeneity among individuals.

In summary, our findings establish m^6^A reader YTHDF2 as a clinically relevant immune checkpoint in DCs, demonstrating its role in impaired antigen presentation, radiotherapy resistance, and metastatic progression. By uncovering the YTHDF2-Notch-MHC-I axis, we provide new insights into epi-transcriptomic regulation of DC function in anti-tumor immunity. Furthermore, our study highlights the therapeutic potential of targeting YTHDF2 to enhance DC-based cancer vaccines, offering a promising approach to improve radiotherapy outcomes and metastatic control. These results lay the groundwork for the development of next-generation DC immunotherapies, with significant implications for overcoming RT resistance and enhancing anti-tumor immunity.

## Materials and methods

### Cancer cell lines

Murine colon adenocarcinoma cell (MC38), Lewis lung carcinoma (LLC), and melanoma cells (B16F10) were obtained from American Type Culture Collection (ATCC). B16F10-OVA, B16F10-SIINFEKL(OT-I)-Zsgreen (B16F10-OZ) and Hct-116 cells were preserved in our lab. Pancreatic cancer cells (KPC344) were kindly gifted from Hidayatullah G Munshi’s lab in Northwestern University.(Pham et al., 2022) These cell lines were cultured in Dulbecco’s modified Eagle’s medium in supplementation with 10% fetal bovine serum and 1% Penicillin Streptomycin. All cell lines were maintained in a humidified incubator at 37°C under 5% CO_2_.

### Mice models

All mice were housed and used according to the animal experimental guidelines set by the Institute of Animal Care and Use Committee (IACUC) of The University of Chicago. All C57BL/6 mice were purchased from Harlan Envigo. *Cd11c*^cre^, and OT-I transgenic mice were purchased from The Jackson Laboratory. *Ythdf2*^flox/flox^ mice were generated using CRISPR-Cas9 technology as described.(Li et al., 2018a) DC-specific *Ythdf2* knockout mice were generated by crossing *Ythdf2*^flox/flox^ mice with *Cd11c*^Cre^ mice (*Ythdf2*-cKO). All mice were maintained under specific pathogen-free conditions and female mice with age at 8–12 weeks were used in accordance with the animal experimental guidelines.

### Human samples

Clinical PBMCs were obtained from patients with metastatic NSCLC enrolled in a clinical trial at our institution (COSINR study, NCT03223155); patients were treated with sequential or concurrent SBRT and immune checkpoint blockade therapy (Ipilimumab and Nivolumab). PBMCs were collected prior to treatment and following completion of SBRT. The procedure for human sample collection was approved by the University of Chicago Biological Sciences Division IRB, and written informed consent was obtained from all patients. All experiments were performed in compliance with the Helsinki Declaration. HLA-typed PBMCs from healthy donors (donor ID# 888360769) was obtained from STEMCELL for human CD8^+^ T cell isolation and DC induction.

### Tumor growth and treatment

One million MC38, LLC, B16F10-OZ or B16-OVA tumor cells were subcutaneously injected in the right flank of mice. Mice were pooled and divided into different groups at random when the tumor volume reached 100 mm^3^. 20 Gy dose of irradiation were delivered to local tumors-localized radiation. For CD8^+^ T cell depletion experiments, 200 μg of anti-CD8a antibody were delivered by intraperitoneal injection, start from one day before other treatments (twice a week). For DC vaccine treatment, BMDCs cocultured with YTHDF2 inhibitor (10 μg/mL) were injected intratumorally. For orthotopic tumor models, 5 × 10^4^ KPC cells was inoculated to the pancreas of mice and followed by treatment with IR (6 Gy) on day 11 under guidance of CT imaging. Mice were measured according to the IACUC requirement, and tumor volumes were calculated by volume = length × width × height × 0.5. Animals were euthanized when the tumor length reached 20 mm, ulceration length > 10 mm, or in poor health state in compliance with IACUC guidelines.

### Flow cytometry

Tumors were collected, diced into small pieces, and digested with digesting medium containing 1 mg/ml collagenase and 200 μg/ml DNase I. Cell suspensions were obtained through a 70 μm cell strainer and washed with flow cytometry staining (FACS) buffer which were made with PBS containing 2% FBS and 0.5 mM EDTA. Dead cells were stained with Zombie-NIR. Cells were blocked with anti-FcR for 10 min and subsequently stained with surface antibodies for 30 min at 4 °C in the dark. For intracellular staining of IFN-γ, cells were stimulated with Cell Activation Cocktail with Brefeldin A for 6–8 h and followed by fixation and permeabilization using Thermo FoxP3 Fixation and Permeabilization Kit before intracellular staining. For intracellular staining of YTHDF2, fixed DCs were incubated with the YTHDF2 antibody at 4 °C overnight, followed by adding the Alexa Flour 647 goat anti-rabbit IgG (Life technologies) and staining for 1 h. Samples were tested with Cytek Aurora benchtop analyzer at Flow Cytometry Core facility of The University of Chicago and the generated data was analyzed by FlowJo software.

### Induction of bone-marrow derived dendritic cells

Single-cell suspensions of bone marrow cells were collected from WT and *Ythdf2*-cKO mice. Red blood cells were removed with Ammonium-Chloride-Potassium (ACK) buffer. Then cells were cultured in a humidified incubator at 37°C and 5% CO_2_ with Iscove’s modified Dulbecco’s medium (IMDM) containing 10% FBS, 1% penicillin streptomycin solution, 55 mM 2-mercaptoethanol, 1% sodium pyruvate, 1% MEM non-essential amino acid, and 100 ng/ml of FLT3L. Equal volume of fresh media containing FLT3L was added on day 5, and bone-marrow derived dendritic cells (BMDCs) were collected on day 9 for further applications.

### Human dendritic cell induction

To induce human DCs, CD14^+^ monocytes were isolated from human PBMCs using EasySep^™^ Human CD14 Positive Selection Kit II. The isolated CD14^+^ cells were cultured with RPMI1640 medium which contains 10% FBS, 1% penicillin streptomycin solution, 1% L-Glutamine solution, human GM-CSF (1000 U/mL), and human IL-4 (250 U/mL). On day 3, fresh medium was added and cells were cultured for another 48 h. Immature DCs were treated with YTHDF2 inhibitors for 24 h (10 μg/mL) and further incubated with EBV peptide or irradiated EBV-positive Hct116 cells to get antigen-loading DCs. Thereafter, DCs were activated with TNF-α for 24 h before coculture with human CD8^+^ T cells for antigen-cross presentation assays. Human CD8^+^ T cells were isolated from PBMCs using MojoSort^™^ Human CD8^+^ T Cell Isolation Kit according to the manufacturer’s protocol.

### *In vivo* migration of DC

The migration of DCs in vivo was performed as previously describes(Liu et al., 2019). Briefly, BMDCs were labelled with 0.5 mM of CFSE. 2 million labeled cells was injected intravenously in mice bearing B16F10 tumors after treatment with or without irradiation. Tumors were collected at 24 h after DC administration, the proportion of CFSE labeled DC was measured by flow cytometry.

### Antigen-cross presentation assay

For antigen cross presentation functional assay, 1 × 10^7^ BMDCs were collected with irradiated (40 Gy) or non-irradiated B16F10-OZ cells (ratio = 1:1) overnight. BMDCs were isolated with EasySep Mouse CD11c Positive Selection Kit II. Meanwhile, CD8^+^ T cells were sorted from tumor draining lymph nodes (TDLNs) and spleens of OT-1 mice using EasySep Mouse CD8^+^ T Cell Isolation Kit. 4 × 10^4^ of sorted DCs were cocultured with 2 × 10^5^ CD8^+^ T cells in each well of pre-incubated IFN-γ^+^ ELISPOT plate for 72 h. Following with biotinylated detection antibody incubation and streptavidin-HRP enzyme conjugation, the cytokine spots of IFN-γ were detected with AEC Substrate Set and counted with immuno-spot analyzer. Tumor-infiltrating DCs were sorted from B16F10-OZ tumors at day 5 post-IR for function assays following the above protocols.

### Real-time quantitative polymerase chain reaction (RT-qPCR)

Total RNA was extracted by using RNeasy Plus Mini Kit. cDNA was then synthesized by using High-Capacity cDNA Reverse Transcription Kit. RT-qPCR using SYBR Green PCR Master Mix was performed in QuantStudio 3 according to manufacturer’s instruction. The specific mRNA primers for RT-qPCR are as follows: *Ythdf2* forward 5’-GAGCAGAGACCAAAAGGTCAAG-3’, *Ythdf2* reverse 5’-CTGTGGGCTCAAGTAAGGTTC-3’; *Aph-1c* forward 5’-CTCTCCTGGGTTTCCACGAC-3’, *Aph-1c* reverse 5’-CAAAGAACACAACGCCCCAG-3’, *Mfng* forward 5’-TTCCTCCTTTCCCGTTGGTGT-3’, *Mfng* reverse 5’-GCCCTGTATCATCCTCTGGG-3’, *Aph-1b* forward 5’-CTGGGGCGTTGTGTTCTTTG-3’, *Aph-1b* reverse 5’-AAATGCCCAGATGCCCATGA-3’, *Gm8909* forward 5’-GGTGGTGTTGCAGAGACGCT-3’, *Gm8909* reverse 5’-CTGCTCTTCAACACAAAAGG-3’, *H2-Q6* forward 5’-GTATTTCCACACTGCTGTGTCCT-3’, *H2-Q6* reverse 5’-AAGGACAACCAGAATAGCTACGT-3’, *Gapdh* forward, 5′-AGGTCGGTGTGAACGGATTTG-3′, *Gapdh* reverse, 5′- TGTAGACCATGTAGTTGAGGTCA-3′; *Gapdh* was chosen as an endogenous control. Gene expression was calculated by using the 2-ΔΔCt method, fold difference = 2^–(ΔCtA −ΔCtB)^, where Ct represents cycle threshold.

### Western blotting analysis

Proteins was extracted with RIPA containing protease and phosphatase. Protein concentration was measured by using the Pierce BCA protein assay kit. Equal amounts of proteins were separated by SDS-PAGE and transferred to PVDF membranes. Then, the membranes were blocked in 5% non-fat dry milk and probed with primary antibodies overnight at 4°C. The next day, membranes were incubated with an HRP-conjugated secondary antibody after washed with TBST and finally detected using enhanced chemiluminescence.

### Gene knockdown in BMDCs

SiRNAs targeting *Spi1*, *Batf*, *RelA*, *Aph-1b*, *Aph-1c*, and *Mfng* were purchased from Santa Cruz Biotechnology. SiRNAs was transfected into BMDCs by Genlantis GeneSilencer^®^ siRNA Transfection Reagent according to manufacturer’s procedures. Two days after the transfection, the cells were collected. The knockdown efficiency was detected by qPCR.

### Gene overexpression in BMDCs

For gene overexpression, 293 T cells were seeded into D60 plates and cultured with DMEM medium containing 10% FBS overnight. On day 2, OPTI-MEM containing transfer vector (*Gm8909*-eGFP_MSGV1 or H2-Q6-eGFP_MSGV1), retrovirus packaging vector (pCL-Eco), and polyethylenimine (PEI) were dropwise added to cells to continue culture for 48–72 h. For transduction, supernatant media containing retrovirus were harvested and mixed with protamine sulfate (10 μg/mL) and fresh prewarmed DMEM containing 10% FBS. BMDCs were dispersed in the mixed medium containing retrovirus and spinoculated at 1000 g for 90 min at 32 °C. GFP expression level in DCs was checked with fluorescence microscopy before further assays.

### Notch pathway activation assays

Notch activation assays were conducted using Mouse Notch1 Pathway Reporter Kit. Briefly, 3 × 10^4^ DCs were seeded into each well of clear-bottom 96-well plate and cultured at 37 in a CO_2_ incubator. After 24 h, cells were transfected with medium OPTI-MEM/Lipofectamine 2000 containing CSL (CBF1/RBP-Jk) luciferase reporter, Renilla luciferase and Notch1ΔE vector. Fresh medium was added after 24 hours of transfection. Luciferase assays were performed after two days transfection with TWO-Step Luciferase (Firefly & Renilla) Assay System (BPS Bioscience). The luciferase signals of Firefly or Renilla were read with luminometer.

### Bulk mRNA-seq

Tumor-infiltrating DCs were collected on day 5 post IR and followed by RNA extraction using RNeasy Plus Mini Kit. Cells were FACS-sorted directly into RULT lysis buffer from the QIAGEN RNeasy UCP Micro Kit and total RNA was extracted using the manufacturer’s instructions. mRNA was enriched and RNA-seq libraries were constructed using the Illumina TruSeq Stranded mRNA kit. Paired-end, dual-index sequencing was performed on the Illumina NovaSeq 6000 system.

### RIP-seq

The procedure was adapted from a previous report.^(Han et al., 2019)^ Briefly, DCs were washed twice with cold PBS and the cell pellet was re-suspended with two volumes of lysis buffer (150 mM KCl, 10 mM HEPES pH 7.6, 2 mM EDTA, 0.5% NP-40, 0.5 mM dithiothreitol (DTT), 1:100 protease inhibitor cocktail, 400 U/ml RNase inhibitor). The lysate was incubated on ice for 5 min and centrifuged for 15 min to clear the lysate. One-tenth volume of cell lysate was saved as input and total RNA was extracted using Trizol. The rest of the cell lysate was incubated with 5 μg anti-YTHDF2 (Aviva systems) at 4 °C overnight with gentle rotation followed by incubation with 40 μl protein G beads for 1 h at 4 °C. The beads were then washed five times with 1 ml ice-cold washing buffer (200 mM NaCl, 50 mM HEPES pH 7.6, 2 mM EDTA, 0.05% NP-40, 0.5 mM DTT, 200 U/ml RNase inhibitor). The immunoprecipitation complex was resuspended in 400 μl 1 × Proteinase K and digested with 2 mg Proteinase K at 55 °C for 1 h. RNA was then extracted using an RNA isolation kit (Zymo). Input and immuno-precipitated RNA of each sample were used to generate the library using a Takara SMARTer Stranded Total RNA-Seq Kit v2 - Pico Input Mammalian.

### m^6^A-seq

Total RNA was isolated from DCs using RNeasy Plus Mini Kit. Two cycles of ploy(A) selection were performed to get enriched mRNA. The obtained mRNA was used for m^6^A immunoprecipitation (m^6^A-IP) with the NEB EpiMark N^6^-methyladenosine enrichment kit according to the manufacturer’s protocol. RNA was enriched with Zymo Research RNA Clean & Concentration-5 and followed by library construction using Takara SMARTer Stranded Total RNA-Seq Kit v2 - Pico Input Mammalian. Library sequencing was performed at the University of Chicago Genomics Facility core with an Illumina Novaseq machine in pair-read mode at 100 bp per read.

### RIP-qPCR analysis

RIP for YTHDF2 was performed using 20 mg anti-YTHDF2 rabbit polyclonal antibody (Aviva systems biology), as described above. After IP, RNA was isolated from Input and IP fractions using phenol/chloroform extraction. cDNA was prepared with the Applied Biosystems High-Capacity cDNA Reverse Transcription Kit (Thermo). SYBR-green-based qPCR was performed using QuantiStudio3 (ABI).

### Chromatin immunoprecipitation (ChIP) qPCR Assay

ChIP assays were performed with a Magna ChIP A/G Chromatin Immunoprecipitation Kit in accordance with the manufacturer’s instructions. Briefly, 1×10^7^ BMDCs were treated with irradiated tumor cells for different time points as indicated and fixed with 1% formaldehyde, cross-linked, and sonicated. Cell lysates were incubated with antibody of interest or IgG control antibody and protein A/G magnetic beads overnight at 4°C. The next day, Protein/DNA Complexes were eluted and reversed cross-linked. DNA was purified for RT-qPCR by using the *Ythdf2* promoter DNA-specific primers. Input (1% of the chromatin) was chosen as internal control and the results are shown as the percentage of input (100×2^((Input Ct - 6.64) – *Ythdf2* Ct)^).

### RNA seq analysis

Fastp software (v0.20.0) was used to trim adaptor and remove low quality reads to get high quality clean reads.(Chen et al., 2018) STAR software (v2.7.9a) was used to align the high-quality clean reads to the mouse reference genome (mm39).(Dobin et al., 2012) featureCounts software (v2.0) was used to get the raw gene level mRNA read counts as the mRNA expression profile.(Liao et al., 2013) DESeq2 software (v1.30.1) was used to normalize and calculate the fold change and Pvalue between two groups.(Love et al., 2014) Ensembl GTF gene annotation database (v104) was used to annotate the mRNA. Gene Ontology (GO) and KEGG pathway enrichment analysis were performed with clusterProfiler R package (v3.18.1) based on the differentially expressed mRNAs.(Yu et al., 2012) rMATS software (v4.1.1) was used to predict the alternative splicing events between two groups.(Shen et al., 2014) For mRNA m^6^A MeRIP-seq analysis, MACS2 (version 2.2.7.1) was used for detection m^6^A peaks.(Zhang et al., 2008) R package DiffBind (3.0.15) was used for methylation difference calculation between groups.(Ross-Innes et al., 2012) Peaks on mRNA was annotated by R ChIPseeker (1.26.2) using gtf annotation file (V104) from Ensembl database.(Yu et al., 2015) HOMER (4.11) was employed for m^6^A mRNA peak motif analysis.(Heinz et al., 2010) R Guitar (2.6.0) was utilized for drawing the metaplot of mRNA methylation.(Cui et al., 2016) For YTHFDF2 RIP-seq, input on each GENCODE annotated gene were counted using HTSeq and then differentially expressed genes were called using DESeq2 package.(Love et al., 2014) YTHDF2 target genes were identified as differentially up-regulated genes comparing YTHDF2 IP sample with the corresponding Input samples. Functional enrichment analysis was performed with DAVID.(Sherman et al., 2022)

### Confocal imaging

For DC subcellular localization analysis, cells were co-stained with the following dyes and antibodies: ER-Tracker Red to label the endoplasmic reticulum, LysoTracker Red to stain lysosomes, and CellMask Deep Red to outline the plasma membrane. For detection of β2m, cells were fixed with 4% paraformaldehyde, permeabilized with 0.1% Triton X-100, and incubated with β2m antibody followed by a fluorescently conjugated secondary antibody. Nuclear staining was performed with DAPI. After staining, DCs were then centrifuged onto slides and mounting with Prolong Gold medium to prevent photobleaching. Confocal images were acquired using Leica SP8 laser scanning confocal microscope equipped with a 63× oil-immersion objective. Image analysis and colocalization quantification were performed using ImageJ software.

### Quantification and statistical analysis

To estimate the statistical significance of differences between two groups, we used a paired or un-paired Student’s t-tests to calculate two-tailed P values. One-way analysis of variance (ANOVA) or two-way ANOVA with multiple comparison test was performed when more than two groups were compared. Error bars indicate the standard error of the mean (SEM) and standard deviation (SD). P values are labeled in the figures. P values were denoted as follows: *P< 0.05, ** P< 0.01, *** P< 0.001. Statistical analyses were performed by using GraphPad Prism (version 8.0).

## Supplementary Material

Figure S2 **Figure S2. SPI1 promotes transcription of Ythdf2 in the context of IR. (A)** GO enrichment analysis of upregulated transcription pathways in tumor-infiltrating DCs of IR versus non-IR. **(B)** GO enrichment analysis of downregulated transcription pathways in tumor-infiltrating DCs of IR versus non-IR. **(C)** ChIP-qPCR results showing RELA binding to the promoter region of *Ythdf2* (n = 3). **(D)** ChIP-qPCR results showing BATF binding to the promoter region of *Ythdf2* (n = 3). **(E-G)** ChIP-qPCR results showing SPI1 **(E)**, BATF **(F)**, and RELA **(G)** binding to the promoter region of *Ythdf2* at different time point (n = 3). **(H)** *Ythdf2* mRNA expression in *RelA* KD DCs with or without irradiation (n = 3). (**I**) *Ythdf2* mRNA expression in *Batf* KD DCs with or without irradiation (n = 3). (**J**) mRNA expression of relevant genes in DCs with or without siRNA treatment (n = 3). **(K)** Quantification of SPI1 level in Figure 2F. Statistical analysis was performed using two-sided unpaired Student’s t test (**C**-**D**, **H**-**J**); ns, P>0.05; ***P<0.001. Data are represented as mean ± SEM.

Figure S1 **Figure S1. IR induces YTHDF2 expression in DC. (A)** Schematic illustration of patients’ PBMC from COSINR trial for YTHDF2 detection. Gating strategy of dendritic cells from PBMC of lung cancer patients in COSINR trial. **(B)** DC frequency in CD45^+^ cells of PBMC before IR, n = 14. (**C**) Dot plot of marker gene expression in tumor-infiltrating DC subsets. **(D)** DC frequency in CD45^+^ cells of PBMC post IR, n = 14. (**E**) Proportion of DC subsets from tumors with or without IR treatment. (**F**) YTHDF2 expression in DCs from responder or non-responder patients’ PBMC prior to IR treatment (n = 14). (**G**) Dot plot representing percent expression and average expression of YTHDF2 in DCs pre or post IR. **(H)** UMAP displaying different clusters of tumor-infiltrating dendritic cells with control mice or IR treatment by scRNA-seq analysis. **(I)** Dot plot representing percent expression and average expression of YTHDF2 in different clusters of tumor-infiltrating DCs by control or IR treatment. **(J)** YTHDF2 relative mRNA expression in Flt3l DCs cocultured with MC38-OZ tumors with or without IR, n = 3. **(K)** YTHDF2 MFI in DCs cocultured with MC38-OZ tumor cells which were treated with or without IR, n = 3. **(L)** Western blotting of YTHDF2 protein in Flt3l DCs cocultured with MC38-OZ tumors treated with or without IR. **(M)** Normalized mean grey value (MGV) of western blotting results in Figure 1L. **(N)** YTHDF2 relative mRNA expression in Flt3l DCs directly treated with or without IR, n = 3. **(O**) YTHDF2 mRNA expression DCs cocultured with irradiated tumor cells or supernant of tumor cells culturing medium, n = 3. **(P**) YTHDF2 MFI in different subpopulations of DCs cocultured with irradiated or non-irradiated tumor cells. Statistical analysis was performed using unpaired Student’s t test (B, D, F, J-K, N-P); ns = not significant, P>0.05; **P<0.01; ***P<0.001. Data are represented as mean ± standard error of mean (SEM), and n = number of samples. Source data are available for this figure: SourceData FigS1.

Figure S4 **Figure S4. IR induces YTHDF2 to target Notch signaling in DC. (A)** Venn diagram of overlapping genes from mRNA-seq that were upregulated following downregulated following *Ythdf2*-cKO+IR versus WT+IR and WT+IR versus WT. **(B)** Heatmap of differentially expressed genes in DCs from WT+IR and WT group (left), and heatmap of differentially expressed genes in DCs from cKO+IR in comparison with WT+IR group (right). **(C)** Schematic illustration of Notch signaling pathway. Ligands in signal-sending cells can bind to glycosylated NOTCH receptors on signal-receiving cells. Disintegrin Metalloproteases (ADAMs) are recruited for cleavage of the outside domain of Notch. After cleavage, the remaining part of the NOTCH receptor can be further cleaved on the cell membrane by γ-secretase and transported into lysosomes for generation of notch intracellular domain (NICD). NICD can be translocated into the nucleus to crosstalk with other signaling pathways and regulate transcription. **(D)** Enrichment of *Mfng*, *Aph-1b*, and *Aph-1c* mRNA in the YTHDF2-immunoprecipitated RNA fraction of BMDCs, determined by RIP-qPCR, n = 3. **(E-G)** BMDCs WT and *Ythdf2*-cKO mice and were treated with actinomycin D. mRNA was collected at indicated time points after treatment and mRNA levels of **(E)** *Aph-1b*, **(F)** *Aph-1c*, and **(G)** *Mfng* were measured by RT-qPCR, n = 3. **(H)** mRNA level of *Mfng*, *Aph-1b*, and *Aph-1c* from tumor-infiltrating DCs of WT, WT+IR and cKO, and cKO+IR mice, n = 3. **(I)** mRNA expression level of Notch receptors in WT or cKO DCs that cocultured with irradiated tumor cells, n = 3. **(J)** mRNA expression level of Notch ligands in tumor cells with or without irradiation, n = 3. Statistical analysis was performed using two-sided unpaired Student’s t test (C-H); **P<0.01; ***P<0.001. Data are represented as mean ± SEM.

Figure S3 **Figure S3. YTHDF2 depletion in DC boosts radiotherapy antitumor immunity by enhancing antigen cross-presentation. (A)** B16F10-OZ tumor growth curves in WT and *Ythdf2*-cKO mice with or without IR (n = 5, mean ± SD). **(B)** Survival rate B16F10-OZ inoculating mice with or without IR. **(C)** Scheme of KPC344 orthotopic pancreatic cancer models and treatment of CT imaging guided RT. **(D-I)** Flow cytometry on immune profiles of **(D)** CD4^+^ Tcells, **(E)** CD8^+^ T cells, **(F)** Macrophages, **(G)** cDCs, **(H)** cDC1s, **(I)** cDC2s residential in B16F10-OZ tumors on day 5 post-IR (n = 5, mean ± SEM). (**J**) CSFE^+^ DCs frequency in tumor infiltrating DCs (n = 4, mean ± SEM). (**K-L**) Mean fluoresence intesity of **(K**) CD80 and **(L)** CD86 in BMDCs (n = 3, mean ± SEM). **(M)** Viabiliy of DCs pre- and post cocultured with CD8+ T cells (n = 3, mean ± SEM). **(N)** ELISPOT assay on IFN-γ secreted by CD8^+^ T cells stimulated with YTHDF2 inhibitor treated DCs in coculture with irradiated or non-irradiated tumor cells (n = 4, mean ± SEM). **(O)** ELISPOT assay on IFN-γ secreted by CD8^+^ T cells stimulated with WT or cKO DCs in coculture with irradiated or non-irradiated tumor cells (n = 4, mean ± SEM). **(P)** Mean fluorescence intensity of H2Kb-SIINFEKL in migratory DCs from WT or cKO mice inoculated with B16-OVA tumors (n = 3, mean ± SEM). **(Q)** ELISPOT assay on IFN-γ secreted by CD8^+^ T cells stimulated with migratory DCs in WT or cKO mice inoculated with B16-OVA tumor cells (n = 3, mean ± SEM). **(R)** ELISPOT assay on IFN-γ secreted by CD8^+^ T cells stimulated with WT or cKO DCs cocultrued with different concentration of SIINFEKL (n = 3, mean ± SEM). **(S)** Mean fluorescence intensity of H2Kd in BMDCs cocultured with irradiated 4T1-HA cells (n = 3, mean ± SEM). (**T**) Mean fluorescence intensity of H2Kd-HA tetramer postive CD8^+^ T cells which were stimulated with DCs cocultured with irradiated 4T1-HA cells (n = 3, mean ± SEM). **(U)** B16F10-OZ tumor growth curves on mice with CD8^+^ T cells depletion by αCD8 (200 μg/mouse, twice weekly), starting 1 day before IR (n = 5, mean ± SD). **(V)** H&E staining of lungs collected from LLC-tumor bearing mice 30 days post tumor inoculation, scale bars: 2 mm. Statistical analysis was performed using two-sided unpaired Student’s t test (A, D-U); ns, P>0.05; *P<0.05; **P<0.01; ***P<0.001.

Figure S5 **Figure S5. The loss of YTHDF2 in DC induces *Gm8909* by Notch signaling pathway and enhances DC vaccine efficacy. (A)** ELISPOT assay on IFN-γ secreted by CD8^+^ T cells, which were co-cultured with DCs single or triple knockdown of *Mfng*, *Aph-1b*, and *Aph-1c. DCs* were exposed to irradiated tumor cells prior than CD8^+^ T cells stimulation. **(B)** Quantification on IFN-γ spots in (S5A) (n = 3, mean ± SEM). **(C)** mRNA expression of relevant genes in DCs with or without treatment of siRNA (n = 3, mean ± SEM). **(D)** mRNA expression of relevant genes in DCs with or without using triple siRNAs for gene knockdown (n = 3, mean ± SEM). **(E)** Volcano plot on the differential expressed antigen-presentation relevant genes in DCs of cKO+IR versus WT+IR. (**F-G**) mRNA expression of **(F)** *Gm8909* and **(G)** *H2-Q6* in DCs from WT, WT+IR and cKO, and cKO+IR mice (n = 3, mean ± SEM). **(H)** mRNA expression of *H2-Q6* in WT or cKO BMDCs with or without triple knockdown of *Mfng*, *Aph-1b*, and *Aph-1c*, n = 3. **(I)** mRNA expression of *H2-Q6* in WT or cKO BMDCs with or without treatment of Notch inhibitor DAPT (n = 3, mean ± SEM). **(J)** Quantification on IFN-γ secreted by CD8^+^ T cells in coculture with H2-Q6/GM8909 overexpressed DCs, which were previously treated with DAPT and exposure to irradiated B16F10-OZ cells, n = 3. **(K)** ELISPOT assay on IFN-γ secreted by CD8^+^ T cells, which were co-cultured with WT/DAPT treated DCs overexpressed with *H2-Q6* or *Gm8909*. **(L)** Quantification on IFN-γ spots in (S5K) (n = 3, mean ± SEM). **(M)** H2-Kb expression level in WT or Gm8909 overexpressing DCs (n = 3, mean ± SEM). **(N)** ELISPOT assay on IFN-γ secreted by CD8^+^ T cells stimulated DCs treated by scrambled siRNA (siScramble) and *Gm8909* siRNA (n = 3, mean ± SEM). **(O)** Confocal fluorescence microscopy of DCs with or without coculture with irradiated tumor cells, scale bars: 5 μm. **(P)** Western blot on co-immunoprecipitation of β2m with Gm8909. **(Q)** B16F10-OVA tumor growth curves of mice intratumorally injected with WT and *Ythdf2*-cKO DC vaccines 3 times/week with or without IR (20 Gy) on day 9 (n = 5, mean ± SD). **(R)** B16F10-OVA tumor growth curves of mice intratumorally injected with WT and inhibitor treated DC vaccines 3 times/week with or without IR (20 Gy) on day 9 (n = 5, mean ± SD). **(S)** LLC tumor growth curves of mice intratumorally injected with WT and inhibitor treated DC vaccines 1 time/week with or without IR (20 Gy) on day 10 (n = 5, mean ± SD). (**T**) Lung metastasis in WT mice receiving different treatments as indicated in Fig. S5S, scale bars: 2 mm. **(U)** Size of lung metastases was measured in Fig. S5T (n = 5, mean ± SEM). **(V)** Size of lung metastases was measured in Fig. 7D (n = 5, mean ± SEM). **(W)** B16F10-OVA tumor growth curves of mice intratumorally injected with inhibitor treated cKO DC vaccines 3 times/week in combination with or without IR (20 Gy) on day 9 (n = 5, mean ± SD). **(X)** MFI of H2Kb-SIINFEKL tetramer^+^ CD8^+^ T cells stimulated with DCs from different treatment (n = 4, mean ± SEM). Statistical analysis was performed using two-sided unpaired Student’s t test (B-D, F-J, L-N, Q-S, U-X); ns, P>0.05; *P<0.05; **P< 0.01; ***P<0.001. Source data are available for this figure: SourceData FigS5.

Table S1

Online supplemental material

Fig S1 shows that radiotherapy induces YTHDF2 in dendritic cells. Fig S2 shows that SPI1 promotes the transcription of *Ythdf2* in dendritic cells in the context of IR. Fig S3 shows that YTHDF2 depletion in DC boosts radiotherapy antitumor immunity by enhancing antigen cross-presentation. Fig S4 shows that IR induces YTHDF2 to target Notch signaling in DC. Fig S5 show that the loss of YTHDF2 in DC induces *Gm8909* by Notch signaling pathway and enhances DC vaccine efficacy.

## Data Availability

The scRNA-seq datasets have been deposited in the Gene Expression Omnibus (GEO) under the accession number GSE206387. Bulk mRNA-seq, RIP-seq, and m6A-seq datasets have been deposited in the Gene Expression Omnibus (GEO) under the accession number GSE309228. All deposited data are publicly available as of the date of publication. This paper does not report original code. Source data are provided with this paper. Any additional information required to reanalyze the data reported in this paper is available from the lead contact upon request.

## Abbreviation list:

RT: radiotherapy
IR: ionized radiation
DC: dendritic cell
BMDC: bone marrow derived dendritic cell
MDSC: myeloid derived suppressing cell
GM-CSF: granulocyte-macrophage colony-stimulating factor
YTHDF: YTH domain-containing family
m6A: N6-methyladenosine
ICB: immune checkpoint blockade
TIME: tumor immune microenvironment
PBMC: peripheral blood mononuclear cell
SBRT: stereotactic body radiotherapy
NSCLC: non-small cell lung cancer
COSINR: concurrent or sequential Ipilimumab, Nivolumab, and stereotactic Body Radiotherapy
MHC-I: Major Histocompatibility Complex class I
PVDF: polyvinylidene fluoride
IP: immunoprecipitation
ChIP: chromatin immunoprecipitation
KD: knock down
cKO: conditional knockout
ICI: immune checkpoint inhibitor
CFSE: 5-(and-6)-carboxyfluorescein diacetate succinimidyl ester
tSNE: t-distributed Stochastic Neighbor Embedding
NICD: Notch 1 intracellular domain
GSEA: gene set enrichment analysis
β2M: beta-2-microglobulin
EBV: Epstein-Barr Virus
MC38: murine colon adenocarcinoma cell
MC38-OZ: MC38-OT1-zsgreen (MC38-OZ)
LLC: Lewis lung carcinoma
ACK: Ammonium-Chloride-Potassium
IMDM: Iscove’s modified Dulbecco’s medium
TDLN: tumor draining lymph node
RIP: RNA immunoprecipitation
DTT: dithiothreitol
s.c.: subcutaneously
