# Supplementary material for: Radiotherapy induces YTHDF2 in dendritic cells impairing cross-presentation and T cell function
Source: J Exp Med. Author manuscript; Available in PMC 2026 Feb 22. (PMC12924711; doi:10.1084/jem.20250641)
Supplement: Supplementary file 1 [file NIHMS2147621-supplement-Figure_S1.pdf]

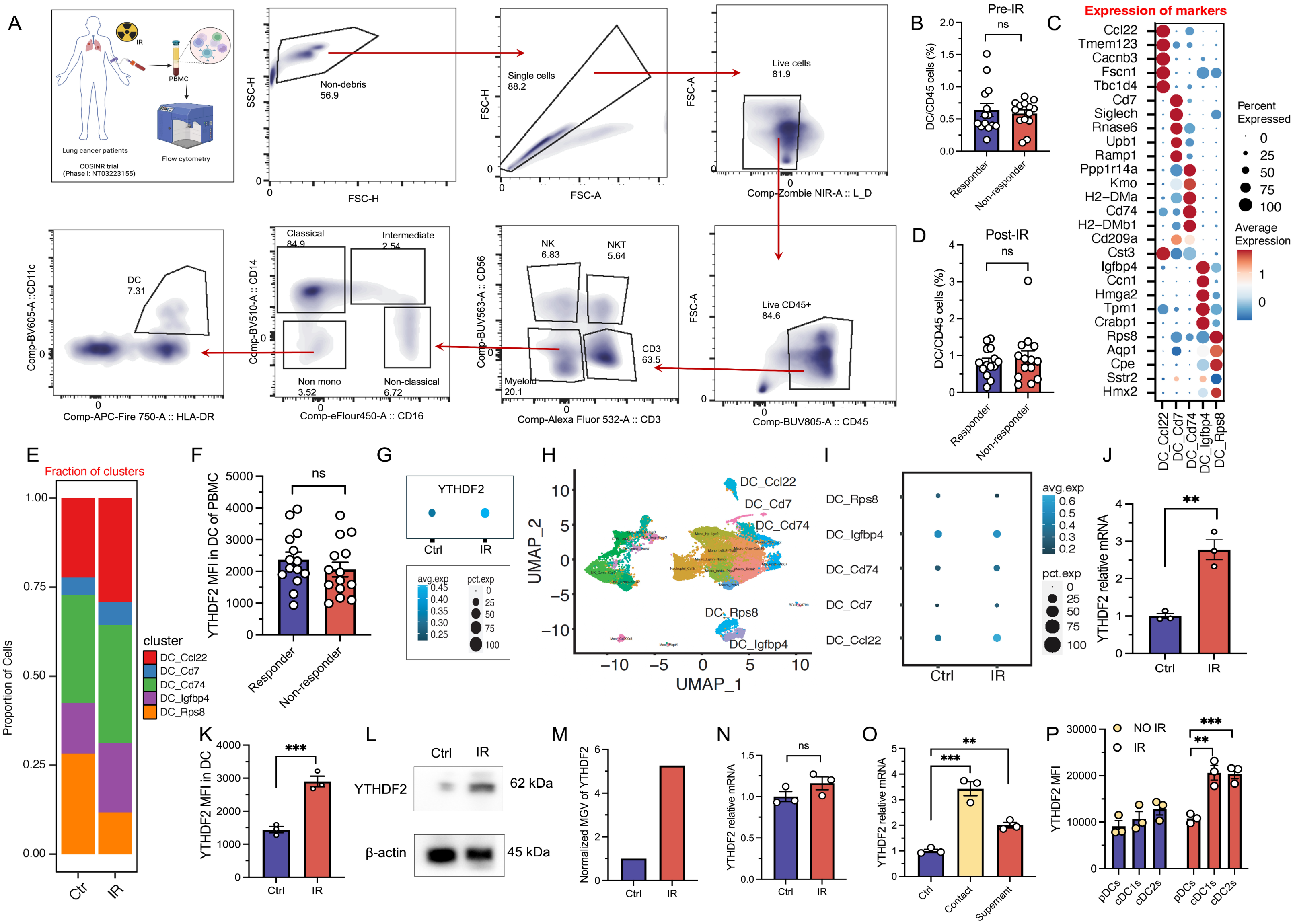

A
Lung cancer patients
COSINR trial (Phase I: NT03223155)
PBMC
Flow cytometry
IR
Non-debris 56.9
Single cells 88.2
Live cells 81.9
Live CD45+ 84.6
CD3 63.5
NK 6.83
NKT 5.64
Myeloid 20.1
Classical 84.9
Intermediate 2.54
Non mono 3.52
Non-classical 6.72
DC 7.31
SSC-H
FSC-H
FSC-A
Comp-Zombie NIR-A :: L_D
Comp-BUV805-A :: CD45
Comp-Alexa Fluor 532-A :: CD3
Comp-BUV563-A :: CD56
Comp-eFlour450-A :: CD16
Comp-BV510-A :: CD14
Comp-APC-Fire 750-A :: HLA-DR
Comp-BV605-A ::CD11c
B
Pre-IR
ns
DC/CD45 cells (%)
Responder
Non-responder
C
Expression of markers
Ccl22
Tmem123
Cacnb3
Fscn1
Tbc1d4
Cd7
Siglech
Rnase6
Upb1
Ramp1
Ppp1r14a
Kmo
H2−DMa
Cd74
H2−DMb1
Cd209a
Cst3
Igfbp4
Ccn1
Hmga2
Tpm1
Crabp1
Rps8
Aqp1
Cpe
Sstr2
Hmx2
DC_Ccl22
DC_Cd7
DC_Cd74
DC_Igfbp4
DC_Rps8
Percent Expressed
Average Expression
D
Post-IR
ns
DC/CD45 cells (%)
E
Fraction of clusters
Proportion of Cells
cluster
DC_Ccl22
DC_Cd7
DC_Cd74
DC_Igfbp4
DC_Rps8
Ctr
IR
F
YTHDF2 MFI in DC of PBMC
ns
Responder
Non-responder
G
YTHDF2
Ctrl
IR
avg.exp
pct.exp
H
UMAP_2
UMAP_1
DC_Ccl22
DC_Cd7
DC_Cd74
DC_Rps8
DC_Igfbp4
I
DC_Rps8
DC_Igfbp4
DC_Cd74
DC_Cd7
DC_Ccl22
Ctrl
IR
avg.exp
pct.exp
J
YTHDF2 relative mRNA
**
Ctrl
IR
K
YTHDF2 MFI in DC
***
Ctrl
IR
L
Ctrl
IR
YTHDF2
62 kDa
β-actin
45 kDa
M
Normalized MGV of YTHDF2
Ctrl
IR
N
YTHDF2 relative mRNA
ns
Ctrl
IR
O
YTHDF2 relative mRNA
***
**
Ctrl
Contact
Supernant
P
YTHDF2 MFI
NO IR
IR
**
***
pDCs
cDC1s
cDC2s
