# Supplementary material for: Radiotherapy induces YTHDF2 in dendritic cells impairing cross-presentation and T cell function
Source: J Exp Med. Author manuscript; Available in PMC 2026 Feb 22. (PMC12924711; doi:10.1084/jem.20250641)
Supplement: Supplementary file 2 [file NIHMS2147621-supplement-Figure_S2.pdf]

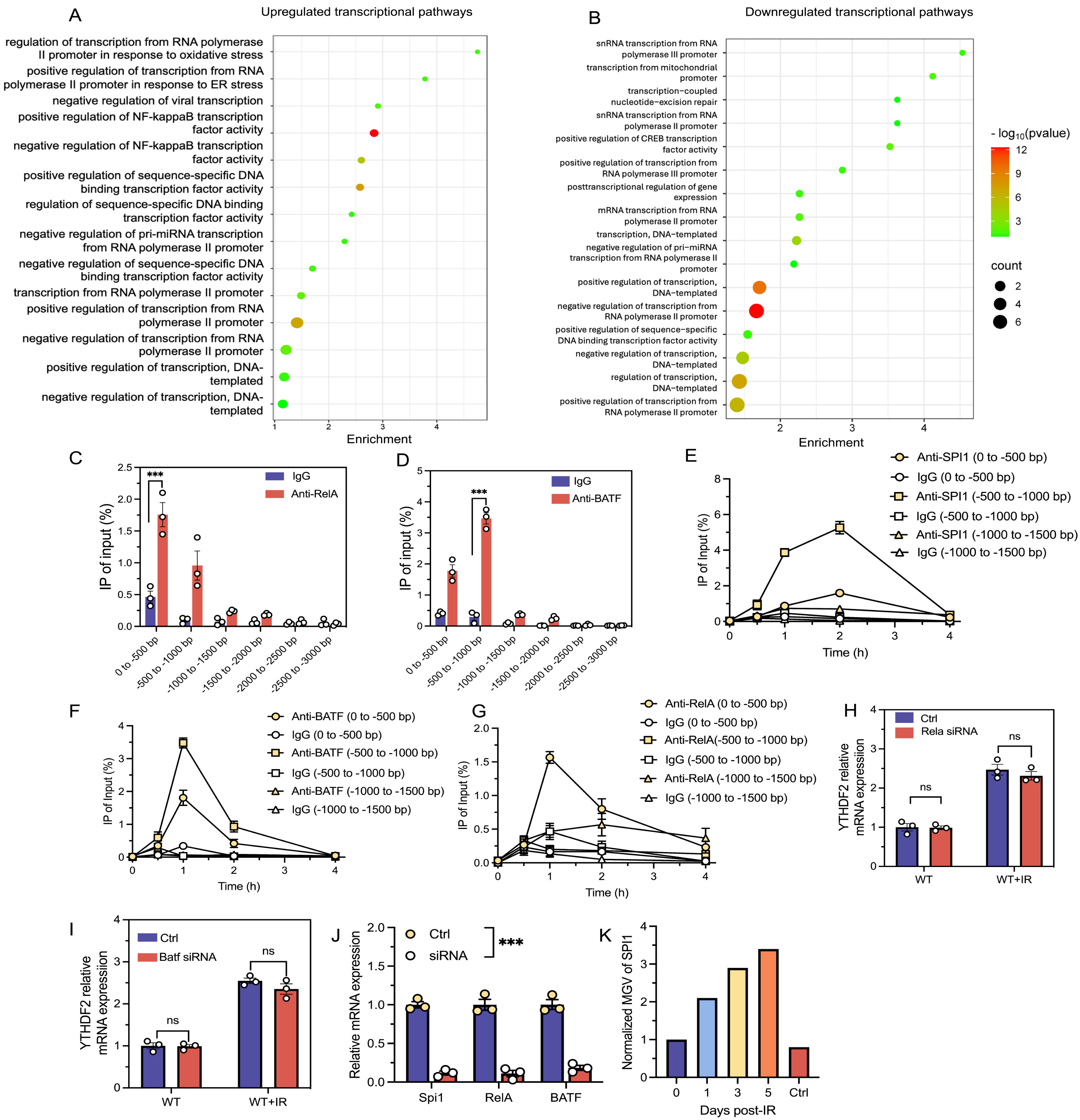
A
Upregulated transcriptional pathways
regulation of transcription from RNA polymerase II promoter in response to oxidative stress
positive regulation of transcription from RNA polymerase II promoter in response to ER stress
negative regulation of viral transcription
positive regulation of NF-kappaB transcription factor activity
negative regulation of NF-kappaB transcription factor activity
positive regulation of sequence-specific DNA binding transcription factor activity
regulation of sequence-specific DNA binding transcription factor activity
negative regulation of pri-miRNA transcription from RNA polymerase II promoter
negative regulation of sequence-specific DNA binding transcription factor activity
transcription from RNA polymerase II promoter
positive regulation of transcription from RNA polymerase II promoter
negative regulation of transcription from RNA polymerase II promoter
positive regulation of transcription, DNA-templated
negative regulation of transcription, DNA-templated
Enrichment
B
Downregulated transcriptional pathways
snRNA transcription from RNA polymerase III promoter
transcription from mitochondrial promoter
transcription−coupled nucleotide−excision repair
snRNA transcription from RNA polymerase II promoter
positive regulation of CREB transcription factor activity
positive regulation of transcription from RNA polymerase III promoter
posttranscriptional regulation of gene expression
mRNA transcription from RNA polymerase II promoter
transcription, DNA−templated
negative regulation of pri−miRNA transcription from RNA polymerase II promoter
positive regulation of transcription, DNA−templated
negative regulation of transcription from RNA polymerase II promoter
positive regulation of sequence−specific DNA binding transcription factor activity
negative regulation of transcription, DNA−templated
regulation of transcription, DNA−templated
positive regulation of transcription from RNA polymerase II promoter
Enrichment
- log10(pvalue)
12
9
6
3
count
2
4
6
C
IP of input (%)
IgG
Anti-RelA
***
0 to -500 bp
-500 to -1000 bp
-1000 to -1500 bp
-1500 to -2000 bp
-2000 to -2500 bp
-2500 to -3000 bp
D
IP of input (%)
IgG
Anti-BATF
***
0 to -500 bp
-500 to -1000 bp
-1000 to -1500 bp
-1500 to -2000 bp
-2000 to -2500 bp
-2500 to -3000 bp
E
IP of Input (%)
Time (h)
Anti-SPI1 (0 to -500 bp)
IgG (0 to -500 bp)
Anti-SPI1 (-500 to -1000 bp)
IgG (-500 to -1000 bp)
Anti-SPI1 (-1000 to -1500 bp)
IgG (-1000 to -1500 bp)
F
IP of Input (%)
Time (h)
Anti-BATF (0 to -500 bp)
IgG (0 to -500 bp)
Anti-BATF (-500 to -1000 bp)
IgG (-500 to -1000 bp)
Anti-BATF (-1000 to -1500 bp)
IgG (-1000 to -1500 bp)
G
IP of Input (%)
Time (h)
Anti-RelA (0 to -500 bp)
IgG (0 to -500 bp)
Anti-RelA(-500 to -1000 bp)
IgG (-500 to -1000 bp)
Anti-RelA (-1000 to -1500 bp)
IgG (-1000 to -1500 bp)
H
YTHDF2 relative mRNA expression
Ctrl
Rela siRNA
ns
ns
WT
WT+IR
I
YTHDF2 relative mRNA expression
Ctrl
Batf siRNA
ns
ns
WT
WT+IR
J
Relative mRNA expression
Ctrl
siRNA
***
Spi1
RelA
BATF
K
Normalized MGV of SPI1
Days post-IR
0
1
3
5
Ctrl
