# Supplementary material for: Radiotherapy induces YTHDF2 in dendritic cells impairing cross-presentation and T cell function
Source: J Exp Med. Author manuscript; Available in PMC 2026 Feb 22. (PMC12924711; doi:10.1084/jem.20250641)
Supplement: Supplementary file 3 [file NIHMS2147621-supplement-Figure_S3.pdf]

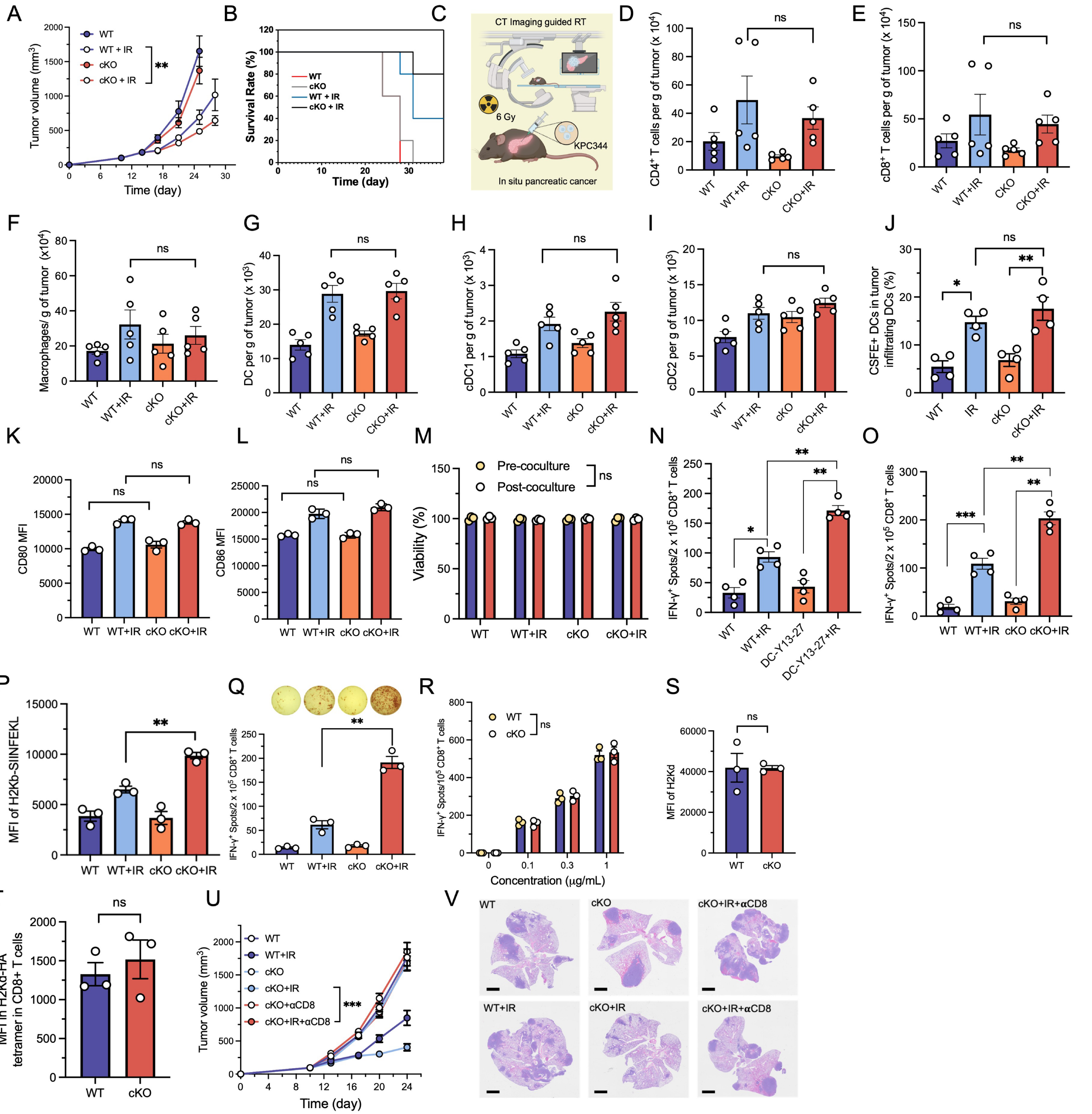
A
Tumor volume (mm3)
Time (day)
WT
WT + IR
cKO
cKO + IR
**
B
Survival Rate (%)
Time (day)
WT
cKO
WT + IR
cKO + IR
C
CT Imaging guided RT
6 Gy
KPC344
In situ pancreatic cancer
D
CD4+ T cells per g of tumor (x 10^4)
ns
WT
WT+IR
CKO
CKO+IR
E
cD8+ T cells per g of tumor (x 10^4)
ns
F
Macrophages/ g of tumor (x10^4)
ns
G
DC per g of tumor (x 10^3)
ns
H
cDC1 per g of tumor (x 10^3)
ns
I
cDC2 per g of tumor (x 10^3)
ns
J
CSFE+ DCs in tumor infiltrating DCs (%)
ns
*
**
WT
IR
cKO
cKO+IR
K
CD80 MFI
ns
L
CD86 MFI
ns
M
Pre-coculture
Post-coculture
ns
Viability (%)
N
IFN-γ+ Spots/2 x 10^5 CD8+ T cells
WT
WT+IR
DC-Y13-27
DC-Y13-27+IR
O
IFN-γ+ Spots/2 x 10^5 CD8+ T cells
***
P
MFI of H2Kb-SIINFEKL
**
Q
IFN-γ+ Spots/2 x 10^5 CD8+ T cells
R
IFN-γ+ Spots/10^5 CD8+ T cells
WT
cKO
ns
Concentration (μg/mL)
S
MFI of H2Kd
ns
T
MFI in H2Kd-HA tetramer in CD8+ T cells
ns
U
Tumor volume (mm3)
Time (day)
WT
WT+IR
cKO
cKO+IR
cKO+αCD8
cKO+IR+αCD8
***
V
WT
cKO
cKO+IR+αCD8
WT+IR
cKO+IR
cKO+IR+αCD8
