# Supplementary material for: Radiotherapy induces YTHDF2 in dendritic cells impairing cross-presentation and T cell function
Source: J Exp Med. Author manuscript; Available in PMC 2026 Feb 22. (PMC12924711; doi:10.1084/jem.20250641)
Supplement: Supplementary file 4 [file NIHMS2147621-supplement-Figure_S4.pdf]

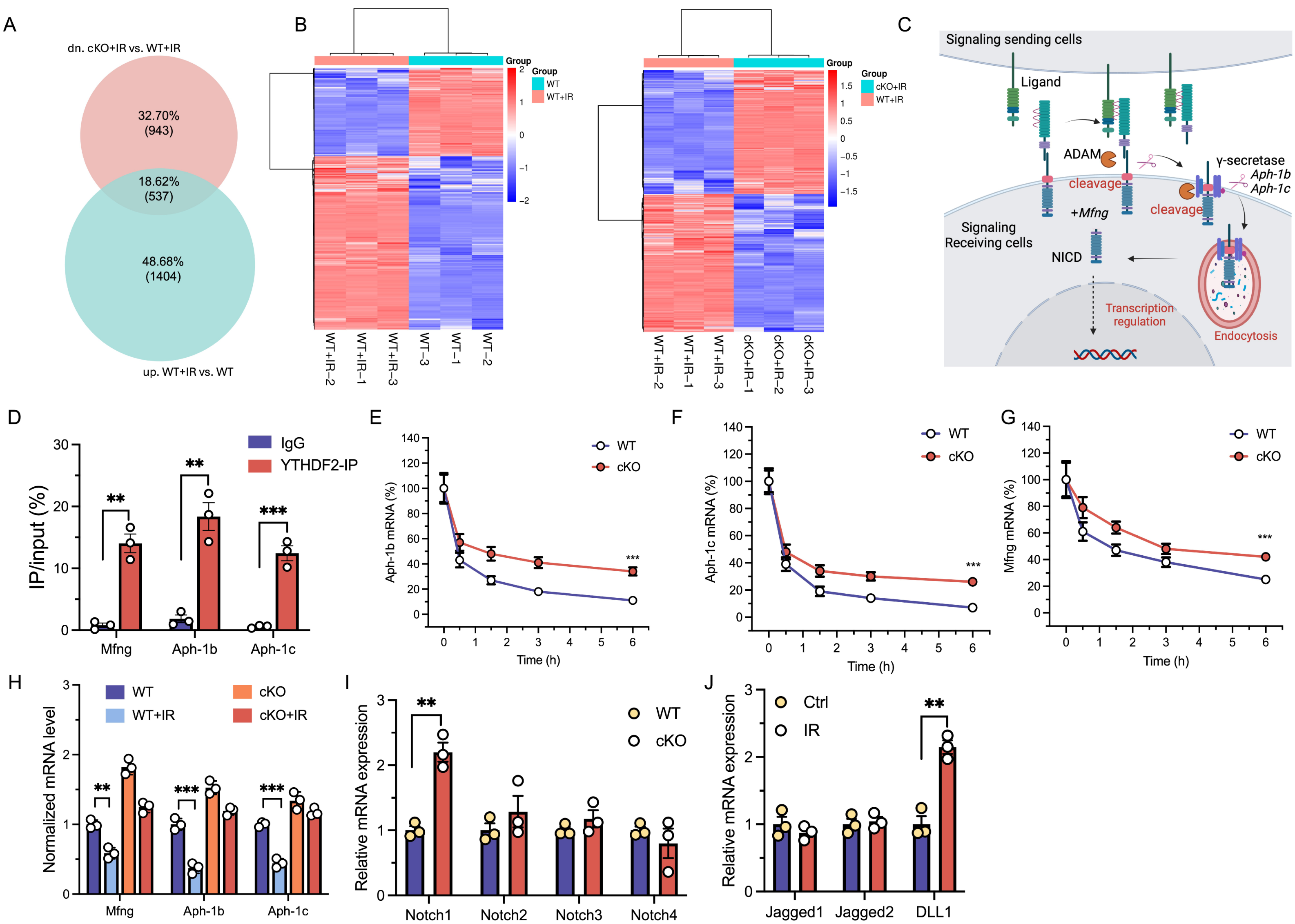

A
dn. cKO+IR vs. WT+IR
32.70% (943)
18.62% (537)
48.68% (1404)
up. WT+IR vs. WT
B
Group
WT
WT+IR
WT+IR−2
WT+IR−1
WT+IR−3
WT−3
WT−1
WT−2
cKO+IR
cKO+IR−1
cKO+IR−2
cKO+IR−3
C
Signaling sending cells
Ligand
ADAM
cleavage
γ-secretase
Aph-1b
Aph-1c
+Mfng
Signaling Receiving cells
NICD
Transcription regulation
Endocytosis
D
IP/input (%)
IgG
YTHDF2-IP
Mfng
Aph-1b
Aph-1c
E
Aph-1b mRNA (%)
Time (h)
WT
cKO
F
Aph-1c mRNA (%)
G
Mfng mRNA (%)
H
Normalized mRNA level
WT
WT+IR
cKO
cKO+IR
I
Relative mRNA expression
Notch1
Notch2
Notch3
Notch4
J
Ctrl
IR
Jagged1
Jagged2
DLL1
