# Supplementary material for: Radiotherapy induces YTHDF2 in dendritic cells impairing cross-presentation and T cell function
Source: J Exp Med. Author manuscript; Available in PMC 2026 Feb 22. (PMC12924711; doi:10.1084/jem.20250641)
Supplement: Supplementary file 5 [file NIHMS2147621-supplement-Figure_S5.pdf]

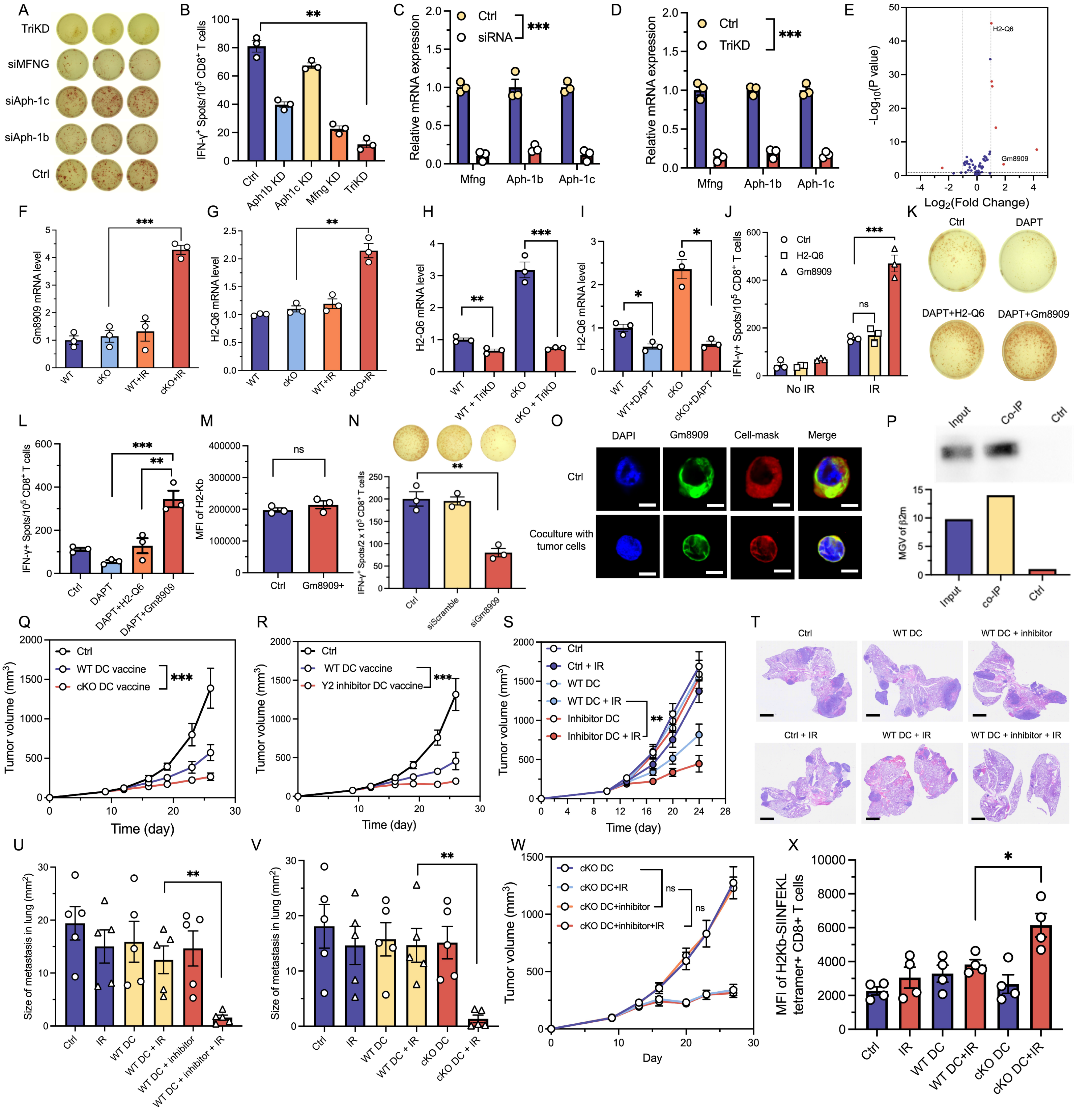

A
TriKD
siMFNG
siAph-1c
siAph-1b
Ctrl
B
IFN-γ+ Spots/10^5 CD8+ T cells
Ctrl
Aph1b KD
Aph1c KD
Mfng KD
TriKD
C
Relative mRNA expression
Ctrl
siRNA
Mfng
Aph-1b
Aph-1c
D
Ctrl
TriKD
E
-Log10(P value)
Log2(Fold Change)
H2-Q6
Gm8909
F
Gm8909 mRNA level
G
H2-Q6 mRNA level
H
I
J
IFN-γ+ Spots/10^5 CD8+ T cells
H2-Q6
Gm8909
No IR
IR
K
Ctrl
DAPT
DAPT+H2-Q6
DAPT+Gm8909
L
M
MFI of H2-Kb
Gm8909+
N
siScramble
siGm8909
O
DAPI
Gm8909
Cell-mask
Merge
Coculture with tumor cells
P
Input
Co-IP
MGV of β2m
Q
Tumor volume (mm3)
WT DC vaccine
cKO DC vaccine
Time (day)
R
Y2 inhibitor DC vaccine
S
Ctrl + IR
WT DC
WT DC + IR
Inhibitor DC
Inhibitor DC + IR
T
WT DC + inhibitor
WT DC + inhibitor + IR
U
Size of metastasis in lung (mm2)
V
cKO DC
cKO DC + IR
W
cKO DC+IR
cKO DC+inhibitor
cKO DC+inhibitor+IR
Day
X
MFI of H2Kb-SIINFEKL tetramer+ CD8+ T cells
